# Aging‐affiliated post‐translational modifications of skeletal muscle myosin affect biochemical properties, myofibril structure, muscle function, and proteostasis

**DOI:** 10.1111/acel.14134

**Published:** 2024-03-20

**Authors:** Clara L. Neal, William A. Kronert, Jared Rafael T. Camillo, Jennifer A. Suggs, Tom Huxford, Sanford I. Bernstein

**Affiliations:** ^1^ Department of Biology, Molecular Biology Institute, Heart Institute San Diego State University San Diego California USA; ^2^ Department of Chemistry and Biochemistry San Diego State University San Diego California USA

**Keywords:** aging, *Drosophila melanogaster*, myofibril, myosin, post‐translational modification, proteostasis, skeletal muscle

## Abstract

The molecular motor myosin is post‐translationally modified in its globular head, its S2 hinge, and its thick filament domain during human skeletal muscle aging. To determine the importance of such modifications, we performed an integrative analysis of transgenic *Drosophila melanogaster* expressing myosin containing post‐translational modification mimic mutations. We determined effects on muscle function, myofibril structure, and myosin biochemistry. Modifications in the homozygous state decreased jump muscle function by a third at 3 weeks of age and reduced indirect flight muscle function to negligible levels in young flies, with severe effects on flight muscle myofibril assembly and/or maintenance. Expression of mimic mutations in the heterozygous state or in a wild‐type background yielded significant, but less severe, age‐dependent effects upon flight muscle structure and function. Modification of the residue in the globular head disabled ATPase activity and in vitro actin filament motility, whereas the S2 hinge mutation reduced actin‐activated ATPase activity by 30%. The rod modification diminished filament formation in vitro. The latter mutation also reduced proteostasis, as demonstrated by enhanced accumulation of polyubiquitinated proteins. Overall, we find that mutation of amino acids at sites that are chemically modified during human skeletal muscle aging can disrupt myosin ATPase, myosin filament formation, and/or proteostasis, providing a mechanistic basis for the observed muscle defects. We conclude that age‐specific post‐translational modifications present in human skeletal muscle are likely to act in a dominant fashion to affect muscle structure and function and may therefore be implicated in degeneration and dysfunction associated with sarcopenia.

## Graphical abstract

Human skeletal muscle myosin heavy chain undergoes aging‐affiliated post‐translational modifications. Mutating the affected residues in the Drosophila model yields defects in myosin biochemistry and cellular proteostasis, as well as dominant negative effects on myofibril structure and muscle function that are enhanced during aging. [Drosophila photo credit: Ian Wright.]

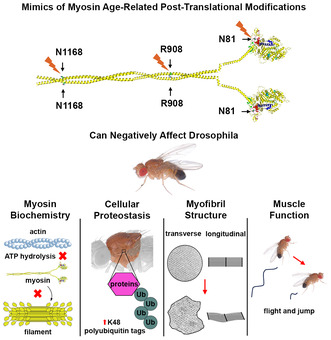

**Abbreviations**
ATPase: adenosine triphosphatase
*fln*
: flightin gene
IFM: indirect flight muscle
*Mhc*
: myosin heavy chain gene
PDB: Protein Data Base
PTM: post‐translational modification
*UAS*
: upstream activation sequence

## 1 INTRODUCTION

During aging, increasing abnormalities in metabolism and mitochondrial function result in accumulation of reactive oxygen species (Damiano et al., 2019; Scicchitano et al., 2018). These contribute to post‐translational modifications (PTMs) of proteins that can be detrimental to their structural and/or functional roles (Baumann et al., 2016). Effects of PTMs arising during muscle aging are further exacerbated by a decline in protein synthesis (Short et al., 2004, although this is controversial, for example, Miller et al., 2019). This may be linked with reduced protein turnover, resulting in failure to replace defective protein. Abnormalities in protein function and accumulation affect proteostasis, the regulated maintenance of the proteome, upsetting the balance of cellular metabolism and performance during the aging process (Fernando et al., 2019).

Skeletal muscles are particularly prone to accumulation of reactive species during aging, due to substantial mitochondrial content necessary to fulfill their high‐energy demands (Boengler et al., 2017). The possibility of age‐specific PTMs is further enhanced for the molecular motor protein myosin as a result of its decreased synthesis rate during aging (Balagopal et al., 1997), coupled with its slow rate of turnover (Papageorgopoulos et al., 2002). A structural and/or functional decline in myosin, which is an integral part of the sarcomere involved in muscle contraction, may be causative of the decreased muscle mass and strength that is observed in sarcopenia, the natural aging process of muscle.

Previously, Li et al. (2015) assessed the presence of aging‐specific PTMs in human skeletal muscle myosin. They found three clusters of such modifications: one in the N‐terminal SH3 domain of the globular motor, a second in the S2 hinge (which positions the motor to interact with actin), and a third in the rod (which forms myosin heavy chain dimers that multimerize to form thick filaments). These investigators determined that myosin from aged humans showed reduced ability to translocate actin filaments in vitro, although they found no significant decline in myosin‐based force production. Further, X‐ray analysis of isolated fibers suggested a less ordered arrangement of filaments in aged individuals. Although PTMs were mapped to the myosin population, they could not be implicated directly in the biochemical or structural effects observed within muscle fibers. Indeed, other factors affecting myosin function or proteins other than myosin could be causative of the perturbations observed in the human study.

Here, we directly assess the role of the specific myosin amino acids found to undergo PTMs in human skeletal muscles by performing an integrative analysis in the *Drosophila melanogaster* model. In contrast to the study involving aged human myosin, we are able to determine whether a particular myosin modification affects muscle structure or function, the biochemical properties of myosin and/or muscle proteostasis. We show that mutations at sites within each of the PTM clusters yield dominant negative effects upon muscle structure and function when compared to a wild‐type myosin control. Further, those in the globular motor domain or S2 hinge reduce in vitro motility and/or ATPase activity, while a PTM in the rod affects myosin filament formation and muscle proteostasis. Our direct demonstration that specific myosin‐based PTM mimics cause defective myosin and muscle abnormalities strengthens the contention that sarcopenia can arise from PTMs in the myosin molecule.

## 2 RESULTS

### 2.1 Design, production, and validation of *Drosophila* lines expressing myosin PTM mimics in skeletal muscle

**FIGURE 1:**
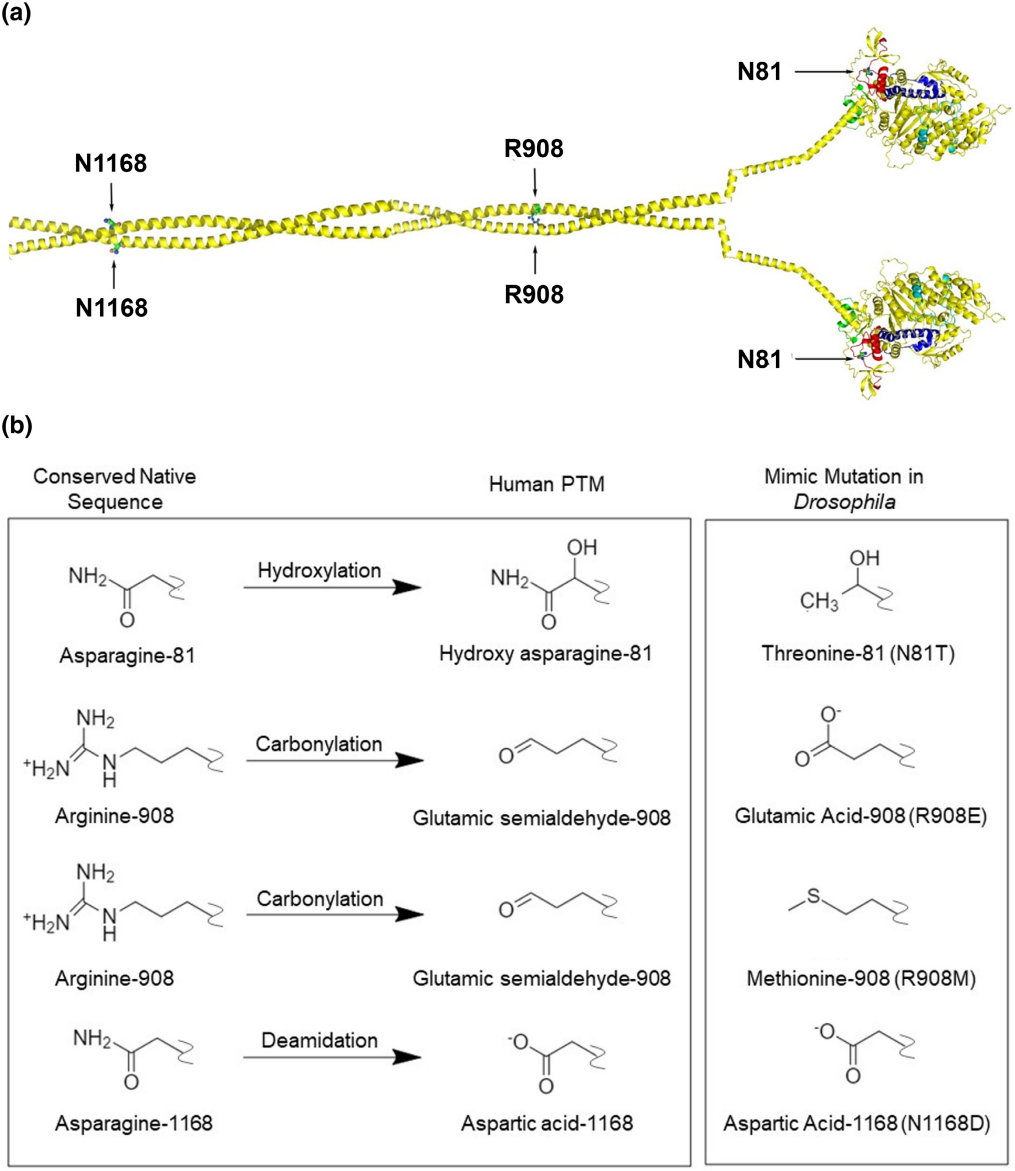
Location of aging‐specific PTMs on the myosin heavy chain molecule and structure of PTM mimics. (a) Composite myosin heavy chain dimer showing the N‐terminal globular heads containing residue N81, the myosin S2 region containing R908, and the rod region containing N1168. In *Drosophila*, the N81 residue is located in an alternative domain encoded by exon 3 (red), with additional alternative domains encoded by exon 7 (cyan, near the ATP‐binding site), exon 9 (blue, relay domain), and exon 11 (green, converter domain) (Bernstein & Milligan, 1997). The molecular model was constructed by docking Protein Data Base (PDB) 4db1a (MYH7 globular head; chain A), PDB 2fxm (human cardiac S2), and PDB 3jbh (tarantula rod). (b) Chemical features of three PTMs that occur in aging human myosin are mimicked by mutations to the native sequence of *Drosophila* myosin: hydroxylation of asparagine‐81 is mimicked by mutation to threonine, carbonylation of arginine 908 is mimicked by glutamic acid or methionine, and deamidation of asparagine 1168 is mimicked by aspartic acid.

We examined the locations of the PTM modifications previously identified in human skeletal muscle myosin heavy chain (Li et al., 2015) and compared the affected amino acids to the sequence deduced from *D. melanogaster* muscle myosin heavy chain gene (*Mhc*), which encodes all isoforms via alternative RNA splicing (George et al., 1989). We chose one conserved amino acid in each previously identified cluster (Li et al., 2015) to mutate for functional analysis—asparagine 81 in the globular head, arginine 908 in the S2 hinge, and asparagine 1158 in the rod (for ease of comparison, we use the human amino acid numbering). The structure of the myosin heavy chain molecule with the location of these amino acids is depicted in Figure 1a.

We designed mutations to test the importance of each side chain and to investigate the effects of some of the specific chemical functional groups contained within the PTMs that had been determined by mass spectrometry (Li et al., 2015). For assessing the importance of each side chain, an alanine was substituted at each site. Additionally (Figure 1b), asparagine 81 that is hydroxylated during aging (personal communication by J. Bergquist) was mutated to threonine (N81T). This models hydroxylation at the beta‐carbon atom. Arginine 908 (loss of guanidino group and carbonylation during aging) was substituted with carboxyl‐containing glutamic acid (R908E) or with neutral methionine (R908M), the side chain of which is isosteric to the glutamic semialdehyde PTM but lacks the ability to accept hydrogen bonds. Asparagine 1168 (deamidated during aging) was replaced with aspartic acid (N1168D), the deamidated form of asparagine.

Transgenic *Drosophila* lines expressing these mutated myosin proteins under the endogenous *Mhc* promoter or the inducible upstream activation sequence (*UAS*) promoter were produced, and the transgenes were expressed in the *Mhc* ^*10*^ genetic background, which lacks endogenous myosin in the indirect flight muscles (IFM) and jump muscles (Collier et al., 1990). For each line, the expected *Mhc* isoform and mutation were validated via sequencing of PCR products of reverse‐transcribed RNA extracted from upper thoraces, which are largely composed of IFM and jump muscles. The levels of myosin heavy chain proteins present in these tissues were assessed by gel electrophoresis (Table 1). Lines with levels of myosin accumulation similar to control were chosen for functional, structural, and biochemical analyses.

**TABLE 1:** Transgenic lines, location, and myosin expression levels.

Line name	Chromosome location	Protein accumulation Mean ± SEM
*pwMhc2*	X	1.00
*pwMhcN81A‐4*	X	0.88 ± 0.01
*pwMhcN81A‐2b*	3	0.91 ± 0.01
*pwMhcN81A‐5b*	3	0.99 ± 0.02
*pwMhcN81A‐9b*	3	0.91 ± 0.01
*pwMhcN81T‐3*	3	0.95 ± 0.00
*pwMhcN81T‐7*	3	0.92 ± 0.01
*pwMhcN81T‐8*	3	0.93 ± 0.01
*pwMhcR908A‐2*	3	0.97 ± 0.01
*pwMhcR908A‐3*	3	0.96 ± 0.01
*pwMhcR908E‐4*	3	0.98 ± 0.00
*pwMhcR908E‐5*	3	0.98 ± 0.00
*pwMhcR908E‐8*	3	0.98 ± 0.00
*pwMhcR908M‐2*	3	0.95 ± 0.00
*pwMhcR908M‐8*	3	0.95 ± 0.01
*pwMhcR908M‐9*	3	0.93 ± 0.00
*pwMhcN1168A‐1*	3	0.93 ± 0.00
*pwMhcN1168A‐2*	3	0.96 ± 0.00
*pwMhcN1168A‐3*	3	0.95 ± 0.01
*pwMhcN1168D‐1*	3	0.93 ± 0.00
*pwMhcN1168D‐2*	3	0.92 ± 0.01
*pUASattB‐Mhc*	3	0.56 ± 0.02
*pUASattB‐N81T‐1*	3	0.54 ± 0.02
*pUASattB‐N81T‐2*	3	0.50 ± 0.01
*pUASattB‐R908E‐1*	3	0.52 ± 0.02
*pUASattB‐N1168D‐1*	3	0.47 ± 0.02
*pUASattB‐N1168D‐2*	3	0.47 ± 0.01
*fln,Mhc* ^*10*^ */fln,Mhc* ^*10*^	2	0.09 ± 0.01

### 2.2 Homozygous expression of alanine mutations and myosin PTM mimics can severely affect muscle function and structure

**FIGURE 2:**
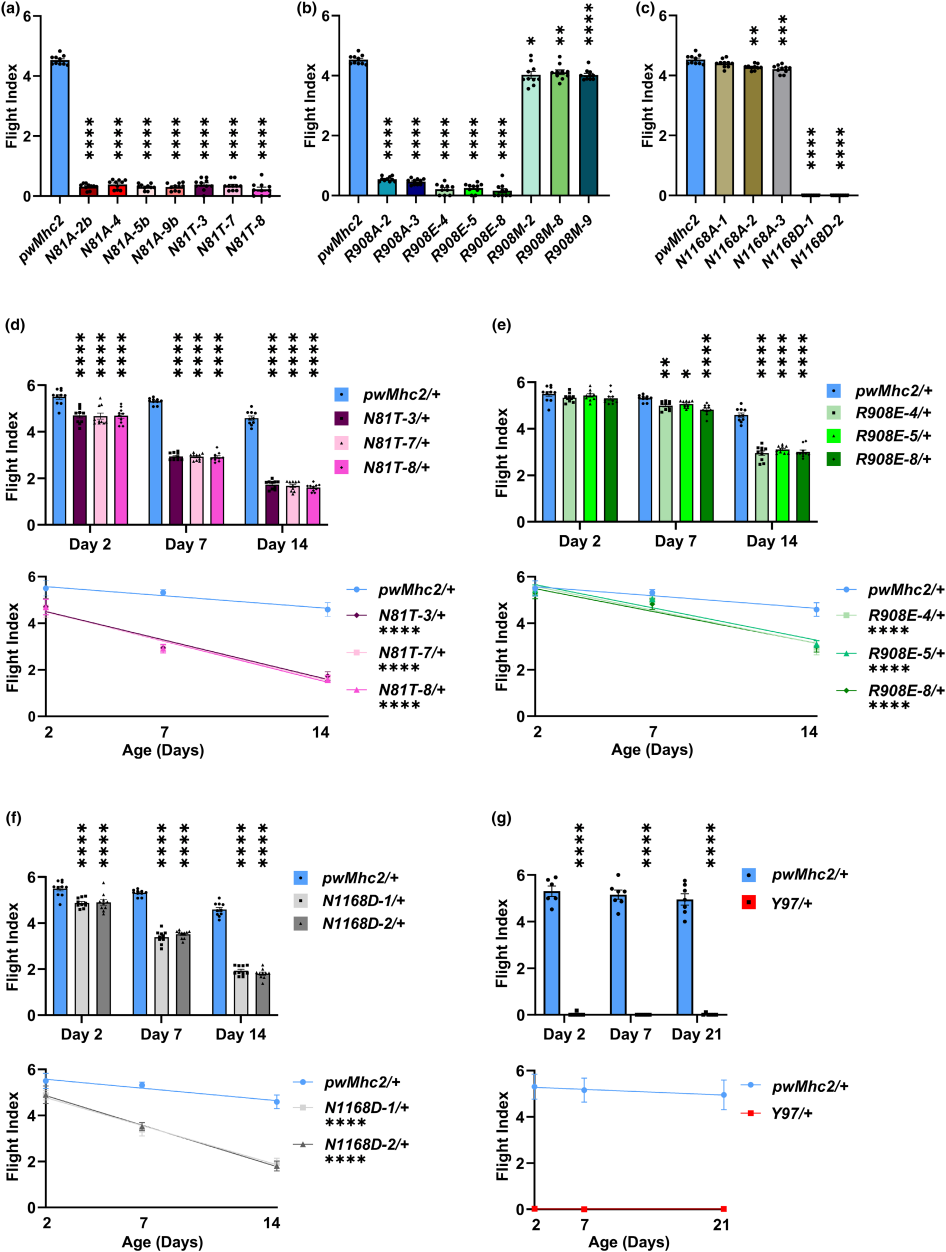
Flight muscle function is compromised in *Drosophila* with mutations in myosin residues that undergo PTM during human aging. (a) Homozygous lines with a deleted side chain of myosin residue N81 (N81A) or containing a hydroxylation mimic (N81T) show dramatically reduced flight capability at 2 days of age. (b) Homozygous lines with a deleted side chain of myosin residue R908 (R908A) or mimicking its carbonylation with carboxyl‐containing glutamic acid (R908E) show dramatically reduced flight capability at 2 days of age, whereas substitution with nonpolar methionine (R908M) has relatively minimal effects. (c) Homozygous lines with a deleted side chain of residue N1168 (N1168A) show minimal effects on flight capability at 2 days of age, whereas deamidation of this residue (N1168D) eliminates flight ability. (d) All *N81T*/+ heterozygotes containing a hydroxylation mimic show significantly reduced flight capability at 2, 7, and 14 days of age. Age‐related decline in flight is enhanced relative to the *pwMhc2*/+ control. (e) All *R908E*/+ heterozygotes containing a carbonylation mimic show significantly reduced flight capability at 7 and 14 days of age. Age‐related decline in flight is enhanced relative to the *pwMhc2*/+ control. (f) All *N1168D*/+ heterozygotes containing a deamidated residue show significantly reduced flight capability at 2, 7, and 14 days of age. Age‐related decline in flight is enhanced relative to the *pwMhc2*/+ control. (g) Heterozygotes for a myosin molecule lacking the globular head (*Y97*/+) show no flight capability at any age tested. All values are mean ± SEM (*p < 0.05, **p < 0.01, ***p < 0.001, ****p < 0.0001).

Multiple lines expressing each alanine mutation and PTM mimic were assessed for flight ability at 2 days of age. Flight indexes were determined for several cohorts of female flies from each genotype, where six signifies upward flight, four horizontal flight, two downward flight and zero no flight. While flies expressing wild‐type myosin (*pwMhc2*) showed a flight index of 4.5, multiple *N81A* and *N81T* lines were essentially flightless with a flight index of 0.23–0.39 (Figure 2a). Thus, the asparagine side chain at residue 81 is critical to IFM function and its mutation to threonine, which contains the same hydroxylation as the reported PTM at this position, is highly deleterious. A similar result was observed for *R908A* and *R908E* lines, but the *R908M* mutant showed only minor effects on flight muscle function (Figure 2b). Thus, while the side chain of R908 is essential for IFM function, it appears that the potential for hydrogen bonding by the modified side chain after loss of its guanidino group contributes to the detrimental effects of PTM at this site. Interestingly, for N1168, the alanine mutation showed little effect on flight ability, whereas the N1186D mutation eliminated flight (Figure 2c). It thus appears that removal of the side chain is not critical to IFM function, but altering its charge is.

**FIGURE 3:**
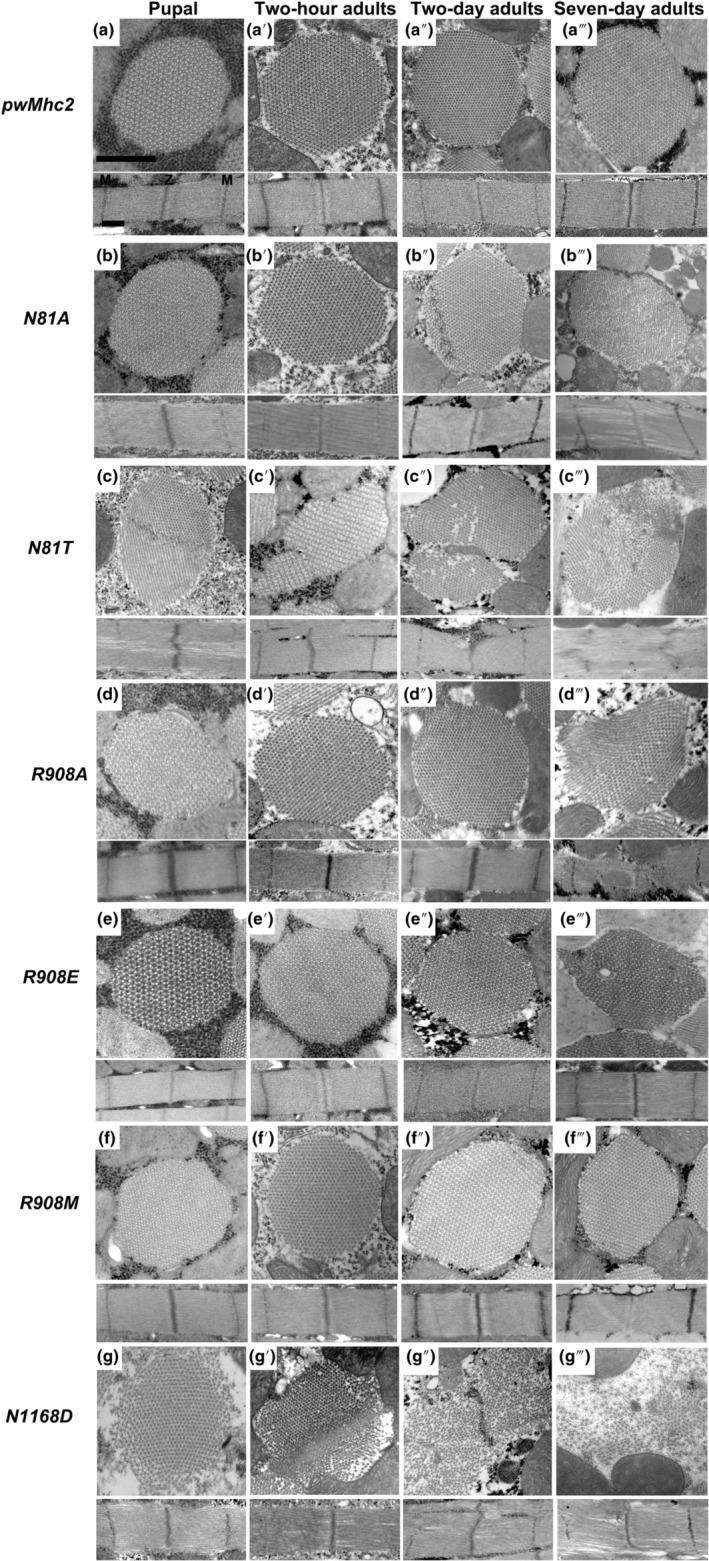
Degeneration of IFMs in *Drosophila* homozygotes for mutations in myosin residues that undergo PTM during human aging. Transverse and longitudinal sections of late‐stage pupae, 2‐hour‐old adults, 2‐day‐old adults, and 7‐day‐old adults were examined by transmission electron microscopy for each genotype. (a–a‴) IFMs from *pwMhc2* containing wild‐type myosin display thick and thin filaments in a double‐hexagonal pattern with regular Z‐ and M‐bands at all stages. (b and b′) *N81A* pupae and 2‐hour‐old adults resemble the control, but (b″ and b‴) two‐ and 7‐day‐old adults show progressive disruptions in thick and thin filament packing and myofibril morphology. (c–c‴) *N81T* late‐stage pupae display assembly defects with frayed sarcomeres containing gaps; severe degeneration occurs during aging such that thick and thin filaments become highly disordered and Z‐ and M‐bands are disrupted. (d–d″) *R908A* organisms appear normal through 2 days of adulthood. (d‴) *R908A* 7‐day‐old adults display myofibril degeneration with disruptions in Z‐ and M‐bands. (e–e″) *R908E* organisms appear normal through 2 days of adulthood. (e‴) *R908E* 7‐day‐old adults display myofibril degeneration with gaps in the filament lattice. (f–f‴) *R908M* IFMs appear normal at all stages. (g) *N1168D* late‐stage pupae display assembly defects with peripheral fraying and wavy sarcomeres. (g′) *N1168D* 2‐hour‐old adults show degenerated myofibrils, with gaps in the sarcomere structure. (g″) *N1168D* 2‐day‐old adult myofibrils display continued degeneration with scattered myofilaments and defects in Z‐ and M‐bands. (g‴) *N1168D* 7‐day‐old adult myofibrils are dramatically disrupted with loosely packed thick and thin filaments; gaps are present in the sarcomere structure, with defects in Z‐ and M‐bands. Z, Z‐band, M, M‐band. Scale bars, 0.55 μm.

We next examined the effects of the homozygous mutations upon IFM myofibril assembly and maintenance using transmission electron microscopy (Figure 3). *pwMhc2* flies expressing wild‐type myosin showed circular myofibrils with a double‐hexagonal array of thick and thin filaments in cross section. Well‐organized sarcomeres were present in longitudinal images. These structures were assembled normally during the pupal stage and maintained through adulthood (Figure 3a–a‴). For N81A, myofibrils appeared normal in pupal and 2‐hour‐old adults (Figure 3b,b′). However, their structure deteriorated somewhat by 2 days and displayed more abnormalities at 7 days (Figure 3b″,b‴). Effects of N81T were more severe, with poor myofibril assembly followed by extreme degradation (Figure 3c–c‴). Both *R908A* and *R908E* lines showed normal assembly and maintenance of myofibrils, with obvious degradation only in 7‐day‐old adults (Figure 3d–d‴,e–e‴). In contrast, *R908M* IFMs maintained normal morphology throughout development and aging (Figure 3f–f‴). For *N1168D*, severe defects in both myofibril assembly and maintenance were obvious, with poor structure at the pupal stage followed by degeneration during aging (Figure 3g–g‴). Overall, the morphological defects at 2 days of age correlate well with the flight defects for the *N81* and *N1168* mutants, but are less severe in the *R908A* and *R908E* lines that also showed defective flight. However, *R908A* and *R908E* IFMs show defects at 7 days, whereas *R908M* (which flies well) appears normal.

We also examined whether the PTM mimics with severe effects upon IFM function affect jump muscle function. We have generally found that mutations in the less‐well‐organized non‐stretch‐activated jump muscle, which contains poorly organized myofilaments, yield less functional perturbation than in stretch‐activated IFM with its cylindrical myofibrils that contain highly organized double‐hexagonal myofilament arrays (Viswanathan et al., 2017) and this appears to be the case for homozygous myosin PTM mimics as well (Figure S1). For this assessment, we performed an aging study to determine whether function declined relative the *pwMhc2* wild‐type control, since defects were not all significant at Day 2. We found that the control line jump distance remained essentially unchanged at Days 2, 7, and 21. However, the values for the PTM mimic mutant flies decreased during aging, with the statistical significance of the average distance generally increased relative to control, particularly for *R908E*. Thus, as for IFM, jump muscles are sensitive to the myosin PTM mimic mutations.

### 2.3 PTM mimics affect myosin ATPase, in vitro actin motility, and/or myosin filament formation

To determine the molecular basis of the defects observed in muscle structure and function that result from expression of the three severe PTM mimics (N81T, R908E, N1168D), we examined the biochemical properties of myosin isolated from their IFMs compared to wild‐type pwMhc2 myosin. For the mutations in or near the globular head (N81T, R908E), we assessed basal and actin‐activated ATPase activity and the ability of the myosin molecules to induce actin sliding in vitro. For the mutations in regions of the molecule that have a propensity to form coiled‐coil dimers (R908E, N1168D), we assessed the ability to form myosin filaments. The mean values and standard deviations/errors for these assays are given in Table 2, with detailed data provided in the Data Supplement.

**TABLE 2:** Biochemical parameters of myosin PTM mimics.

Myosin isoform	*n* for ATPase/motility/EC50	Basal Mg‐ATPase Mean ± SD (s^−1^)	Actin‐stimulated *V*_*max*_ Mean ± SD (s^−1^)	Actin‐stimulated *K*_*m*_ Mean ± SD (μM)	Motility Mean ± SD (μm/s)	EC50 Mean ± SEM (mM)
pwMhc2 (control)	4/3/4	0.10 ± 0.07	1.02 ± 0.19	0.35 ± 0.04	6.65 ± 0.71	160.68 ± 2.02
N81T	2/1/0	None detected	None detected	–	None detected	Not assessed
R908E	4/3/3	0.07 ± 0.03	0.71 ± 0.17*	0.78 ± 0.43	6.29 ± 0.73	155.90 ± 1.23
N1168D	0/0/4	Not assessed	Not assessed	Not assessed	Not assessed	143.90 ± 5.05*

* *p* < 0.05.

We were unable to obtain basal or actin‐activated ATPase activity or in vitro actin motility for N81T myosin, suggesting that the mutation severely affects the function of the motor. For R908E myosin, neither in vitro motility nor basal ATPase activity was significantly different from the control, but actin‐activated ATPase activity was significantly reduced (0.71 ± 0.17 vs. 1.02 ± 0.19 s^−1^; Figure S2). Thus, R908E appears to compromise stimulation of myosin ATPase activity by actin.

To analyze filament formation, we determined how this property varied relative to salt concentration. Myosin filaments form at low salt concentrations, as previously demonstrated by electron microscopy, and they can be differentially pelleted via centrifugation (Viswanathan et al., 2017). For R908E, the percentage of myosin filaments that pelleted via centrifugation at various salt concentrations showed no significant difference from wild‐type in regard to EC50 value (the salt concentration where half of the myosin molecules pellet). However, there was a significant decrease in EC50 for N1168D (143.90 ± 5.05 vs. 160.68 ± 2.02 mM), indicating that it shows a reduced tendency to form filaments compared to wild‐type myosin.

### 2.4 Myosin PTM mimics act dominantly in an age‐specific manner to affect muscle function and structure

As all myosin molecules are not expected to be post‐translationally modified, it is important to determine if the mimic mutations act in a dominant fashion. To examine the effects of the severe PTM mutations on muscle function and structure in the presence of wild‐type myosin, we therefore crossed PTM lines to a wild‐type line to create heterozygotes (mutant/+).

Flight assays for heterozygotes were carried out at 2, 7, and 14 days post‐eclosion to examine aging‐related defects. Effects at Day 2 were much milder than the flightless phenotypes present in homozygotes (Figure 2). While *N81T*/+ and *N1168D*/+ flies showed statistically significant reductions in flight abilities compared to control, *R908E*/+ did not (Figure 2d–f bar graphs). By Day 7, all lines showed significant reductions compared to controls, with further reductions in flight ability seen at Day 14, where the control flight index was 4.6 compared to values of ~1.7 for *N81T*/+, ~3.0 for *R908E*/+ and ~1.9 for *N1168D*/+. Age‐related decline in flight is enhanced in all mutants relative to the *pwMhc2*/+ control (Figure 2d–f line graphs).

An enigmatic aspect of this study relative to the biochemical results (Section 2.3) is that N81T myosin yielded no ATPase activity or actin motility in vitro, yet *N81T*/+ heterozygotes showed flight capability. It is well documented that the presence of a single myosin null allele results in a flightless phenotype (O'Donnell & Bernstein, 1988). We wondered whether the ability of what appears to be a null allele (*N81T*) to allow flight muscle function arises from the fact that its encoded mutant myosin assembles normally into thick filaments with wild‐type myosin. This could permit the latter to interact appropriately to power flight. To test this, we crossed the *Y97* line carrying a headless myosin molecule lacking its motor domain (Cripps et al., 1999) to wild‐type flies and performed flight tests. Despite the headless myosin possessing a rod capable of assembling into thick filaments (Cripps et al., 1999), heterozygous flies were completely flightless (Figure 2g). We speculate on this issue in Section 3.

**FIGURE 4:**
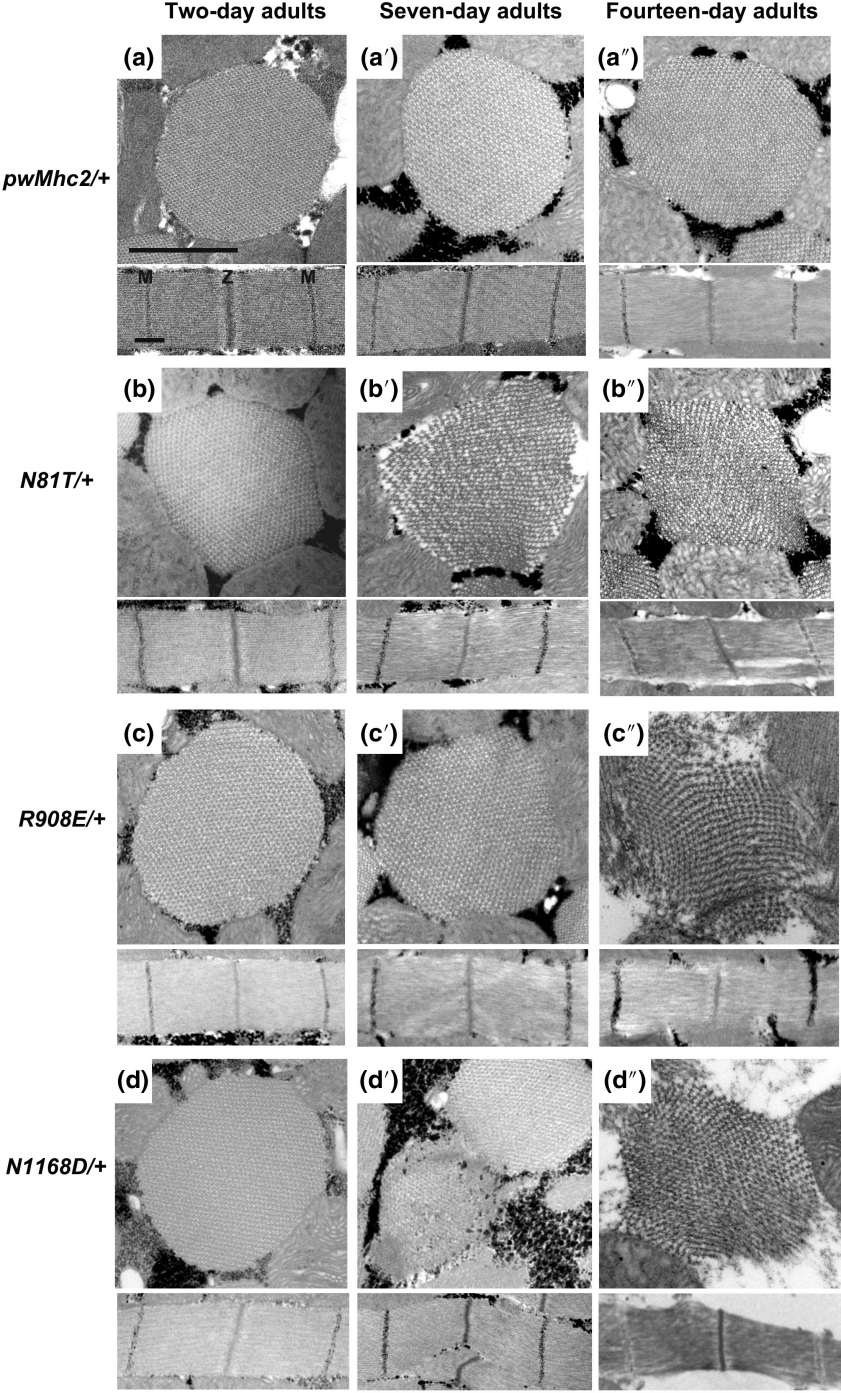
Degeneration of IFM from *Drosophila* heterozygotes for mimic mutations in myosin residues that undergo PTM during human aging. Transverse and longitudinal sections of 2‐day‐old, 7‐day‐old and 14‐day‐old adult IFMs were examined by transmission electron microscopy for each genotype. (a–a″) IFMs from *pwMhc2*/+ adults containing wild‐type myosin display thick and thin filaments in a double‐hexagonal pattern with regular Z‐ and M‐bands at all stages. (b) *N81T*/+ 2‐day‐old adults display wild‐type morphology, but show increasing disarray during aging (b′ and b″), with abnormal myofilament packing and gaps in the sarcomere filament structure. (c and c′) *R908E/+* heterozygotes appear relatively normal through 7 days of adulthood, with some mild perturbations observed in the longitudinal section at 7 days. They show severe degeneration by Day 14 (c″), with disrupted filament packing and gaps in the sarcomere. (d) *N1168D*/+ 2‐day‐old adults display wild‐type morphology, but 7‐ and 14‐day‐old adults show degenerated myofibrils (d′ and d″) with nonlinear sarcomeres. Z, Z‐band, M, M‐band. Scale bars, 0.55 μm.

We next assessed the effects of the heterozygous PTM mimic mutations on the structure of aging IFMs (Figure 4). At 2 days of age, myofibrils from all three mutant heterozygotes showed normal filament packing and sarcomere structures (Figure 4b, 4c, 4d), resembling those of the control (Figure 4a). This is in keeping with the relatively normal flight abilities present at this age (Figure 2d–f). It also illustrates the accumulation of PTM myosin mimic proteins at normal levels, since the wild‐type myosin gene present in the heterozygotes is only capable of producing 50% of normal levels of myosin and expression of a single myosin allele reduces thick filament levels by half (O'Donnell & Bernstein, 1988). While structure is largely retained in 7‐day‐old *R908E*/+ flies (Figure 4c′), the ultrastructures of *N81T*/+ (Figure 4b′) and *N1168D*/+ (Figure 4d′) show serious degeneration, with disrupted filament packing. This increases in severity in 14‐day‐old adults of these genotypes (Figure 4b″,d″) and also occurs in *R908E*/+ IFMs at this age (Figure 4c″), correlating with further reduction in flight ability (Figure 2d–f).

### 2.5 Myosin PTM mimics expressed at lower levels dominantly affect muscle function and structure during old age

To further model the human condition, we expressed each of the severe myosin PTM mimic mutations in a wild‐type genetic background at a later stage of aging by employing the *UAS*‐Gal4 system. Expression of the PTM in a wild‐type background further dilutes the level of the mutant protein, whereas expression later in the life cycle better mimics the aging‐related modifications detected in humans (Li et al., 2015). For this study the *N81T*, *R908E*, and *N1168D* mutant myosin genes were inserted following an inducible *UAS* promoter. To confirm transgene structure and appropriate transcript splicing, a single copy of each transgene was crossed into the *Mhc* ^*10*^ null background along with the IFM‐specific *flightin (fln)* Gal4 driver (http://flybase.org/reports/FBtp0097341). RT‐PCR and DNA sequencing verified correct myosin isoform and mutation expression (see Section 4). Next, levels of myosin in these transgene heterozygotes (one copy of *Mhc* being expressed) were determined by gel electrophoresis and were shown to be at ~50% of homozygous controls (Table 1), indicating that the *fln* driver was about as efficient as the *Mhc* promoter in yielding transgene expression.

**FIGURE 5:**
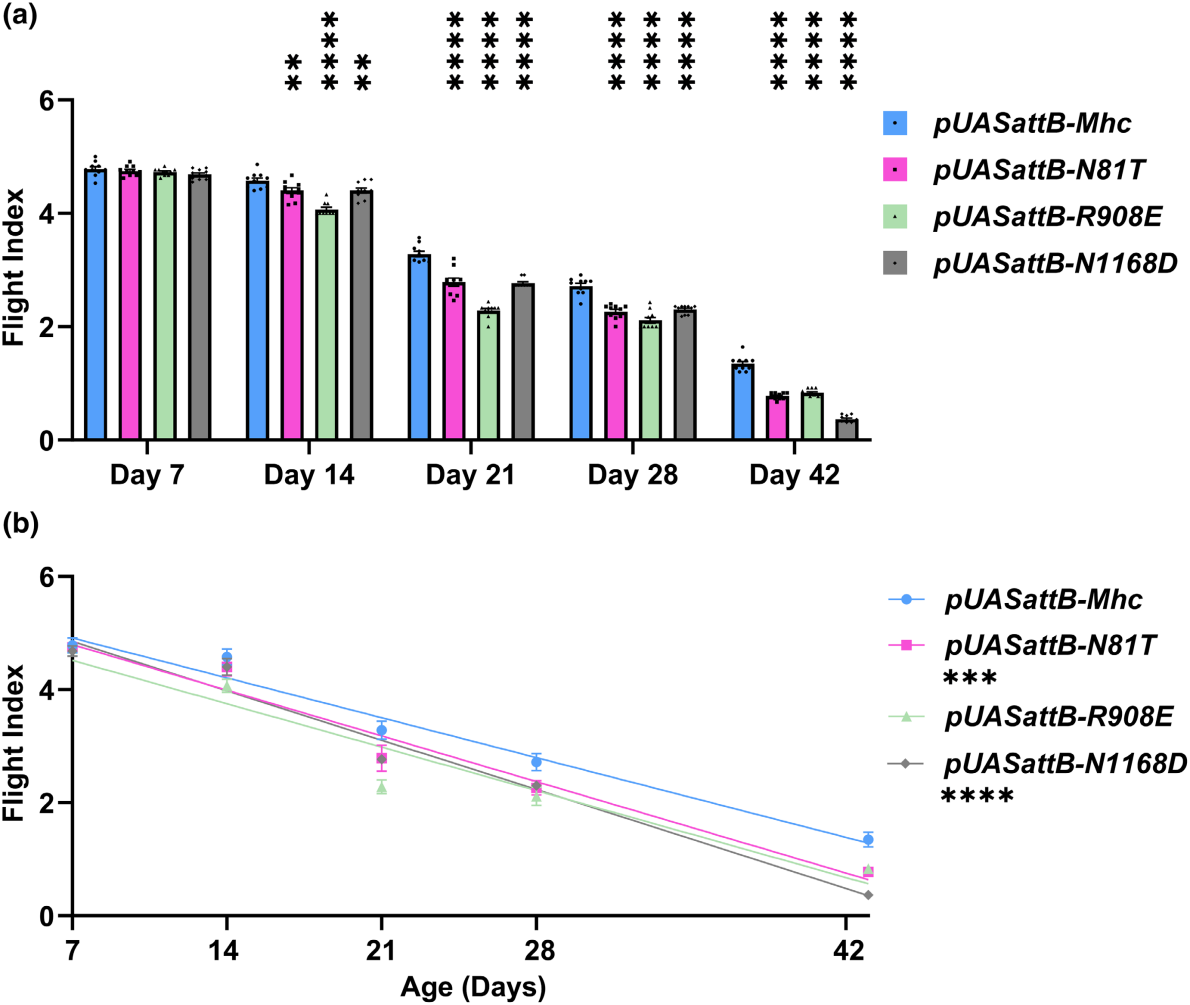
Muscle function is increasingly compromised during aging of wild‐type *Drosophila* also expressing one copy of mimic mutations in myosin residues that undergo PTM during human aging. The *DJ694*‐Gal4 driver, which expresses after eclosion and maintains expression during aging, was used to drive wild‐type (*pUASattB‐Mhc*) or mutant myosin transgenes (*pUASattB‐N81T*, *R908E*, or *N1168D*) via the *UAS* promoter. (a) While *UAS*‐driven expression of PTM mimic myosin in 7‐day‐old adults did not compromise flight ability compared to the wild‐type control, expression in 14‐, 21‐, 28‐, and 42‐day‐old adults led to small but statistically significant decreases in flight ability compared to expression of the wild‐type transgene. (b) Comparison of flight abilities during aging reveals that expression of the *pUASattB‐N81T* or *N1168D* transgenes in a wild‐type background decreases flight significantly more quickly than expression of the wild‐type *pUASattB‐Mhc* transgene. All values are mean ± SEM (**p < 0.01, ***p < 0.001, ****p < 0.0001).

We next employed the *DJ694*‐Gal4 driver encoding EDTP (https://flybase.org/reports/FBti0024387.html) to express each mimic mutation‐containing transgene in the wild‐type background during aging. This driver begins expressing in muscle after eclosion (Bryantsev et al., 2012) and maintains its expression through 50 days of adulthood in IFM (Seroude et al., 2002), at levels similar to *Mhc* (http://flybase.org/cgi‐bin/rnaseqmapper.pl?dataset=celniker_wiggle&xfield1=FBgn0027506 and http://flybase.org/cgi‐bin/rnaseqmapper.pl?dataset=celniker_wiggle&xfield1=FBgn0264695). We measured flight abilities at 7, 14, 21, 28, and 42 days of age. This corresponds to young (7 days), middle age (14–28 days), and aged individuals (42 days), in which flight ability is clearly compromised (Miller et al., 2008). We found that the flight ability of the mimic lines expressing N81T, R908E, and N1168D was no different from a line expressing the wild‐type transgene control at 7 days of age (Figure 5a). However, at 14 days of age, all of the PTM mimic lines showed statistically significant reductions in flight ability compared to control. Flight indexes for both the control and the myosin PTM mimic‐expressing flies progressively worsened from days 14 through 42. Notably, while the PTM mimic lines always showed a significant level of flight reduction compared to the control during the aging process, the rate of reduction during aging was significant for only *N81T* and *N1168D* (Figure 5b). Only *N1168D* showed a significant increase in slope relative to control between Days 21–42 (*p* < 0.0001), indicating a significant reduction in flight ability during this time period.

**FIGURE 6:**
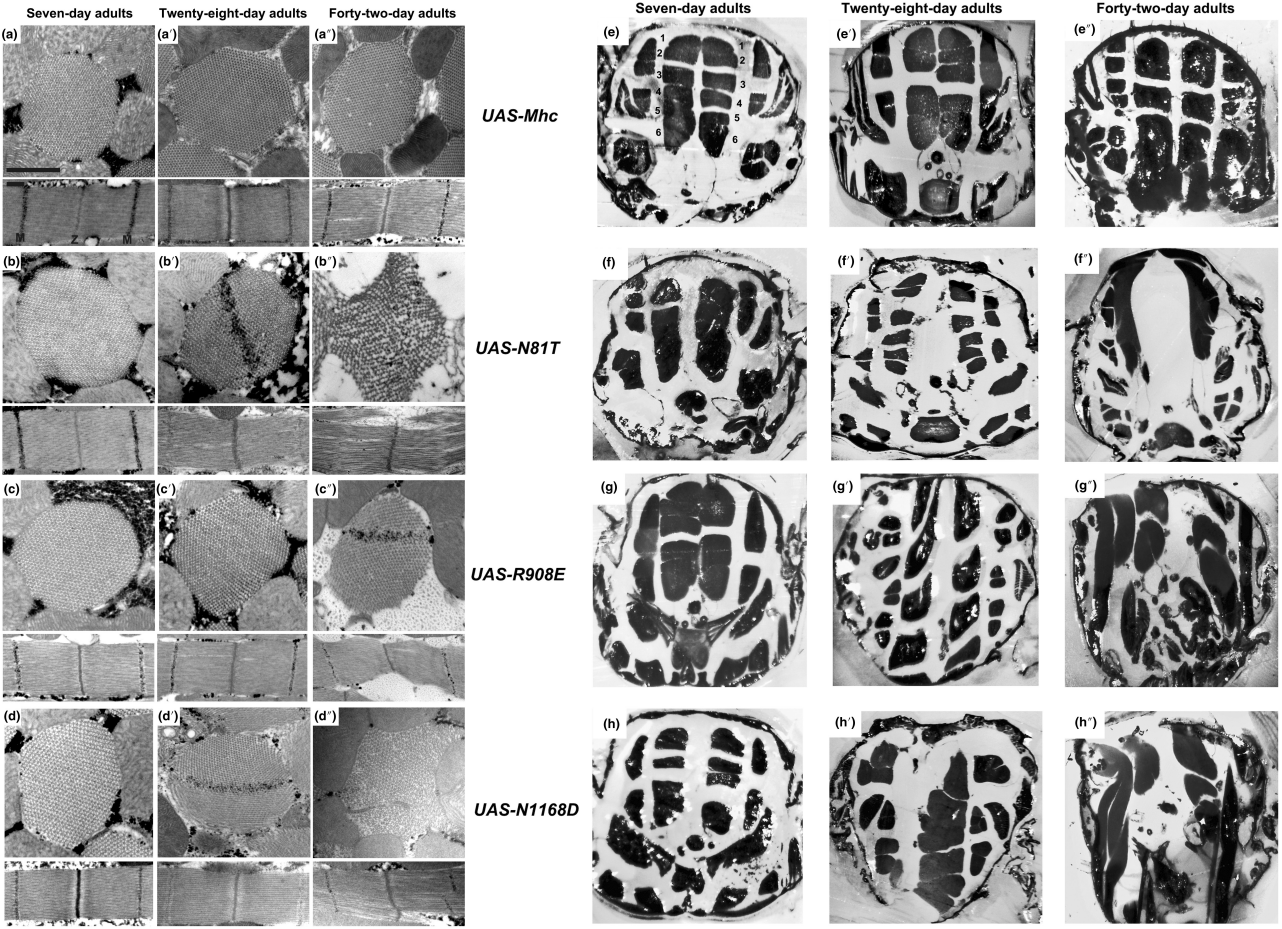
Muscle ultrastructure and fiber integrity are increasingly compromised during aging of wild‐type *Drosophila* expressing one copy of PTM mimic mutations in myosin in a wild‐type background. (a–d″) Transverse and longitudinal sections of 7‐, 28‐ and 42‐day‐old wild‐type adults expressing wild‐type or PTM mimic myosin via the *DJ694*‐Gal4 driver as examined by transmission electron microscopy. Ultrastructure at 7 days for each PTM mimic (b, c, d) appears the same as in wild‐type‐expressing flies (a), mirroring the normal flight abilities at this age (Figure 5). At 28 days, normal structure is retained in flies expressing wild‐type myosin (a′), but flies expressing each of the mutant alleles show degeneration of myofibrillar structure and/or gaps in the sarcomere lattice (b′, c′, d′). At 42 days, flies expressing wild‐type myosin display minor gaps in the myofibrillar lattice (a″), whereas each mutant (b″, c″, d″) shows dramatic abnormalities in myofilament arrangement and sarcomere structure. Z, Z‐band, M, M‐band. Scale bars, 0.55 μm. (e–h″) Cross sections of 7‐, 28‐ and 42‐day‐old wild‐type adults expressing one copy of wild‐type or PTM mimic myosin via the *DJ694*‐Gal4 driver as examined by light microscopy. The dorsolongitudinal IFMs are evident as six fibers on each side of the midline (e). In flies expressing each PTM mutant allele in 7‐day‐old adults (f, g, h), these structures appear similar to those in flies expressing wild‐type myosin (e). However, while this structure is retained in older flies expressing wild‐type myosin (e′ and e″), the shape and size of dorsolongitudinal fibers expressing myosin PTM mimics are clearly abnormal at 28 days (f′, g′, h′) and further degenerate by 42 days (f″, g″, h″). Mutant fibers are often small and displaced, suggesting muscle atrophy.

Electron microscopic analysis of IFMs of the lines expressing the myosin PTM mimics during aging indicated that all lines displayed normal morphology at day 7 (Figure 6a, b, c, d), in keeping with the strong flight abilities shown at this age (Figure 5). However, by 28 days, defects in myofibril morphology with gaps in the sarcomeric structures and abnormal filament orientations were obvious in each of the PTM mimics (Figure 6b′, c′, d′). In contrast, the control line retained wild‐type structure (Figure 6a′). At 42 days, while the wild‐type control line showed minor filament gaps (Figure 6a″), each of the mutant lines displayed extreme filament disorganization, particularly in the cases of *N81T* (Figure 6b″) and *N1168D* (Figure 6d″). Notably, while the control line showed poor flight capability at 42 days, the lines expressing the myosin PTM mimics displayed flight indexes that showed statistically significant reductions in relative flight capability (Figure 5), in keeping with the severe degeneration.

To assess how myosin PTM mimics that are expressed at relatively low levels affect whole muscle morphology, we examined cross sections of aging adult thoraces (Figure 6e–h″). Light microscopy highlights the dorsolongitudinal IFMs, composed of six fibers on each side of the midline. These fibers appear well organized and intact in the line expressing the wild‐type myosin allele at 7, 28, and 42 days of age (Figure 6e–e″). This is also the case for flies expressing *N81T*, *R908E*, and *N1168D* at 7 days of age (Figure 6f, g, h, respectively). However, for the 28‐day‐old thoraces expressing the myosin PTM mimics (Figure 6f′, g′, h′) and particularly for the 42‐day‐old thoraces (Figure 6f″, g″, h″), fibers are dramatically smaller, misaligned and disorganized. It is clear that in addition to ultrastructure, whole fiber morphology is dramatically disrupted by the myosin PTM mimics.

### 2.6 Myosin PTM mimics can disrupt proteostasis

The removal of aggregates of damaged proteins by the autophagy‐lysosome pathway and by the proteasome is critical to maintaining proteostasis in muscle during aging (Combaret et al., 2009). The mechanism for degradation of substrates is dependent on the mode of ubiquitination. Polyubiquitination via linkage at K48 residues (Thrower et al., 2000) typically dictates substrate degradation by the 26S proteasome complex (Glickman & Ciechanover, 2002). However, K48‐linked ubiquitination has also been connected to production of aggregates, particularly those containing mutant proteins, and their possible autophagic elimination (Tan et al., 2008). Thus, K48‐ubiquitin is a reasonable marker to evaluate skeletal muscle protein targeted for degradation in both autophagic and proteasome pathways in response to aging.

**FIGURE 7:**
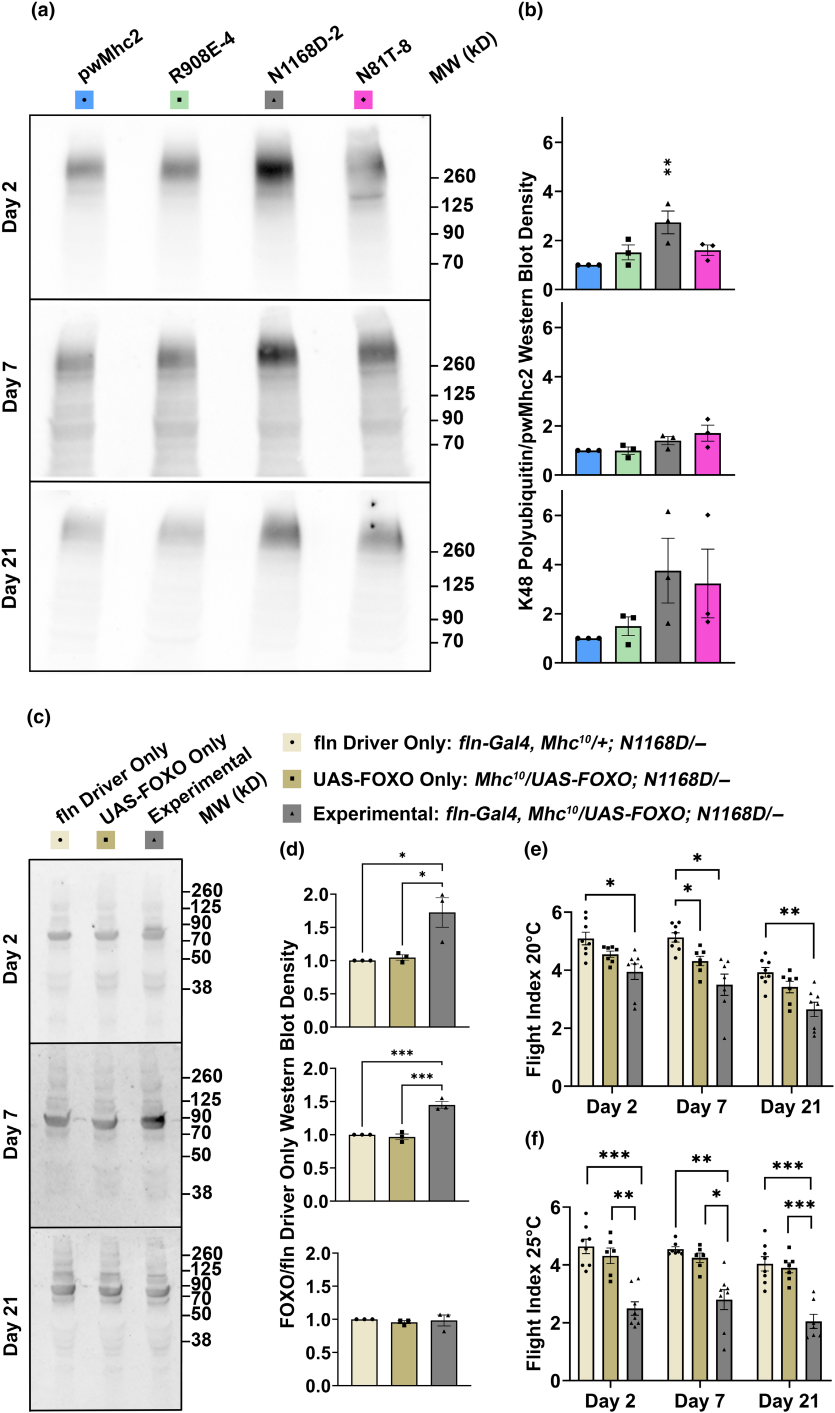
Detection of disrupted proteostasis in *Drosophila* expressing PTM mimic mutations in myosin and assessment of FOXO's ability to ameliorate flight ability dysfunction during aging. (a) Western blots of thoracic proteins from homozygous myosin PTM mimics at 2 days, 7 days, and 21 days probed for K48‐linked polyubiquitin. (b) Quantitative assessment of K48‐linked polyubiquitin relative to total protein accumulated relative to *pwMhc2* control shows that excess ubiquitination in *N1168D* is statistically significant at 2 days. (c) Western blots of thoracic FOXO expression in *N1168D*/+ heterozygotes driven by the *fln* promoter using the Gal4‐*UAS* system (experimental). (d) Quantitation of the blots yields significantly enhanced accumulation of FOXO (between 70 kDa and 90 kDa) in flies with both components of the Gal4‐*UAS* system at 2 and 7 days of age, but not at 21 days of age. Flight testing shows significant reduction of flight ability relative to controls when FOXO is expressed (e) at 20°C or (f) 25°C, at any age tested. All values are mean ± SEM (*p < 0.05, **p < 0.01, ***p < 0.001).

To assess whether the accumulation of K48‐linked ubiquitinated proteins during aging of *Drosophila* muscle is affected by expression of the myosin PTM mimics in the homozygous state, thoracic tissue composed largely of muscle was isolated at Days 2, 7, or 21 for protein extraction and western blotting. Blots were probed with antibody to K48‐linked polyubiquitin (Figure 7a). To compare polyubiquitin accumulation among samples, total protein levels from Ponceau‐stained blots (Figure S3) were quantified by densitometry and used as a normalization factor (Data Supplement). Comparison of ratios of K48‐linked polyubiquitin/total protein between PTM mimics and the pwMhc2 control across aging timepoints is shown in Figure 7b. K48‐linked polyubiquitin significantly increased at 2 days of age in N1168D‐2. The enhanced levels of polyubiquitinated proteins resulting from expression of the N1168D myosin PTM mimic suggest that this mutation disrupts proteostasis and results in overloading of protein degradation pathways, which can arise during aging due to dysfunctional autophagy and/or anomalous proteosome function.

The FOXO transcription factors regulate genes of the autophagy‐lysosomal and proteasomal systems to maintain proteostasis (Demontis & Perrimon, 2010; Kapetanou et al., 2021). Further, an age‐associated decrease in FOXO binding to target genes has been observed in *Drosophila* (Birnbaum et al., 2019). In keeping with these observations, enhanced FOXO expression later in life can ameliorate muscle weakness by enhancing proteostasis (Demontis & Perrimon, 2010). Given the disruption of proteostasis observed from expressing the N1168D myosin PTM mimic (Figure 7a,b), we targeted *N1168D*/+ heterozygotes with enhanced FOXO expression to examine whether this would result in improved flight ability during aging. We first demonstrated enhanced expression of FOXO at Days 2 and 7 via western blotting of *N1168D*/+ thoraces when using the *UAS*‐Gal4 system to express FOXO under the *fln* promoter at 20°C (Figure 7c,d, Figure S3). We then assessed the flight index of FOXO‐overexpressing *N1168D*/+ flies relative to a control lacking the *fln*‐Gal4 driver and a control lacking the *UAS*‐FOXO construct at this temperature. We also employed the enhanced temperature of 25°C for crossing and raising these flies, as it augments *UAS*‐based transgene expression (Duffy, 2002). In contrast to expectations, the experimental flies show reduced flight ability compared to the controls at all of the ages tested (Figure 7e), with the higher temperature yielding more severe effects (Figure 7f).

## 3 DISCUSSION

Abnormal post‐translational modifications of proteins are emerging as important markers of aging (Santos & Lindner, 2017), although their causality of age‐related skeletal muscle weakness is still being established. Abnormal PTMs may arise due to high concentrations of oxidants, which are associated with aging and can impair cross‐bridge kinetics and myofibrillar force generation (Andrade et al., 2001; Persson et al., 2019; Prochniewicz et al., 2008). Notably, while aging‐related changes in the levels of PTMs are beginning to be analyzed (Li et al., 2015), only a few studies have tried to quantify specific PTMs in myosin molecules (Landim‐Vieira et al., 2022; Watanabe et al., 1992).

Oxidant accumulation decreases proteostasis, yielding reduced protein turnover that can cause abnormally modified skeletal muscle proteins to accumulate (Ayyadevara et al., 2016). Aberrant soluble proteins are typically degraded by the proteasome, but once aggregates form, bulk degradation occurs through autophagy (Kocaturk & Gozuacik, 2018). Aging triggers a decline in proteasome function in human muscle, among other tissues (Saez & Vilchez, 2014). Further, autophagic and lysosomal potentials decline during aging because of reduced autophagy inducers, autophagosome components, and cellular responses to hormones (Rubinsztein et al., 2011). Thus, proteins targeted for degradation by addition of ubiquitin may fail to be degraded during aging. Overall, disrupted proteostasis, which includes accumulation of abnormally modified proteins, is likely to be instrumental in yielding the decreased contractile function and muscle mass associated with sarcopenia.

We modeled the effects of aging‐related human myosin PTMs in whole organisms and in vitro, by expressing myosin with key chemical features of these PTMs in the *Drosophila* system. We observed negative effects on IFM and jump muscle function. The severity of effects on IFM structure and function was proportional to the relative level of PTM mimic to wild‐type myosin present. Importantly, these defects were enhanced relative to controls during the aging process. Functional decline during aging likely results from the increasing myofibrillar disarray and fiber degeneration observed in the PTM mimics. This mirrors the less ordered state of skeletal muscle myofibrils detected by X‐ray diffraction of human biopsy samples from aged individuals (Li et al., 2015).

To understand the mechanistic basis of myosin PTM mimic‐induced muscle defects observed in vivo, isolated myosin from each mutant was assessed for its biochemical properties. The N81 residue is absolutely conserved in myosin II molecules and is located near the N‐terminus of the globular motor domain, in a region that acts as a communication pathway between the myosin essential light chain and the nucleotide binding pocket, suggesting it is critical to myosin cross‐bridge kinetics (Bloemink et al., 2020). Hence, it is not unexpected that ATPase activity and in vitro motility were not detected with N81T mutant myosin. This result is somewhat enigmatic, however, given the ability of N81T myosin to participate in IFM function in the heterozygous state (which is not the case for myosin lacking its globular head). It is possible that functional degradation of the protein prior to its isolation from N81T homozygotes, which display severe myofibril degeneration, yielded myosin that was functionally defective due to in vivo degradative processes.

The R908 residue is located in the S2 linker region, which is important in directing interactions of the globular head with actin during the ATPase cycle (Arakelian et al., 2015). Indeed, the trend toward higher actin *K*_*m*_ for R908E myosin suggests reduced actin affinity and this myosin shows a significant decrease in maximal actin‐activated ATPase activity (Table 2). Thus, both N81T and R908E may interfere with cross‐bridge formation, which could reduce stiffness and force generation. R908 also constitutes part of the coiled‐coil domain that is capable of driving heavy chain dimerization. It serves as a “*g*” residue in the heptad repeat, which typically strengthens charge interactions with the “*e*” residue of the partner heavy chain (McLachlan & Karn, 1982). However, we did not find the R908E mutation to affect myosin filament formation. As this residue is located between the light chain binding domain and the region of S2 that is buried in the thick filament (Rahmani et al., 2021), it is unlikely to play an integral role in dimerization and subsequent thick filament formation. Of note, carbonylation of this residue adds a carbon monoxide that can form aldehydes that lack charge or reactive ketones. While we were able to mimic the size and charge distribution that arise from this PTM, we were unable to mimic carbonyl reactivity. However, there is further evidence of the critical nature of this residue in that its mutation to a cysteine in beta‐cardiac myosin has been linked to development of dilated cardiomyopathy (van der Zwaag et al., 2011).

Residue N1168 is an “*a*” residue of the heptad repeat that typically forms a part of the hydrophobic stripe that enhances dimer formation via interaction with a “*d*” residue of the coiled‐coil (McLachlan & Karn, 1982). N1168 is within the thick filament backbone (Rahmani et al., 2021), but it is not hydrophobic. Its transition from an uncharged to a negatively charged form in N1168D myosin might be predicted to disrupt its interaction with its partner “*d*” residue (in keeping with this thesis, substitution with an uncharged A had little effect on muscle function). The N1168D mutation might therefore affect dimer assembly and consequently thick filament assembly or stability. Indeed, we found that decreased salt concentration was necessary to drive the mutant myosin into filaments.

Elevated levels of K48 polyubiquitinated proteins in N1168D suggest that more damaged skeletal muscle proteins accumulate relative to control, with an enhancement in older flies. Based on electron microscopy, abnormal myofibrillar structures increased dramatically during aging of skeletal muscle expressing this myosin PTM mimic. Excessive accumulation of misfolded and/or aggregated proteins that are ubiquitinated for removal may overload the proteasome and autophagic systems and disrupt proteostasis. As FOXO overexpression can significantly downregulate accumulation of ubiquitinated proteins, while upregulating proteasome activity (Manola et al., 2021), we examined if it might combat the effects of PTMs on skeletal muscle function during aging. We found that neither minimal FOXO overexpression at 20°C nor greater FOXO overexpression at 25°C provided a benefit to aging PTM muscle function. In fact, overexpression proved detrimental, which may be due to exacerbation of protein degradation. It is possible that use of other temperature regimens or muscle drivers could yield functional benefits, as FOXO's ability to rescue muscle function can be highly sensitive to dosage (Blice‐Baum et al., 2017; Manola et al., 2021). It is further possible that drug treatment might prove efficacious in improving proteostasis, since drugs such as minocycline can enhance this process through FOXO regulation of Hsp70 and autophagy (Lim & Hyun, 2022). Overall, our PTM models provide direct proof that the modified residues are causative of aging‐related damage to myosin and muscle function, which may yield approaches for discovering therapies for sarcopenia.

## 4 EXPERIMENTAL PROCEDURES

Summaries of methodology are provided here, with details in Supplementary Materials.

### 4.1 DNA constructs

The *Drosophila P*‐element‐containing *Mhc* genomic construct *pwMhc2* was digested with Eag I to produce two subclones. The *pwMhc‐5′* subclone contains an 11.3 kb fragment in *pCasper*. The *pMhc‐3′* subclone contains a 12.5 kb fragment in *pBluescriptKS* (Stratagene, La Jolla, CA). These were substrates for site‐directed mutagenesis following further subcloning. Mutated subclone fragments were sequentially cloned back into the intermediate subclones from which they originated. Mimic mutations were also cloned into the *pUASattB* vector for transgenic insertion using the PhiC31 integrase system.

### 4.2 Production of transgenic lines

Transgenic lines (Table 1) were generated by BestGene, Inc. (Chino Hills, CA) via embryo injection. Transgenes were crossed into the *Mhc* ^*10*^ background, which is null for myosin heavy chain in IFM and TDT muscle (Collier et al., 1990).

### 4.3 Transgenic line validation

RT‐PCR was used to confirm that transgenic *Mhc* transcripts were spliced correctly and contained the site‐directed nucleotide changes. Myosin expression levels relative to actin accumulation were determined for each homozygous transgenic line in an *Mhc* ^*10*^ background by SDS polyacrylamide gel electrophoresis and densitometry. For transgenic *pUASattB* lines, inserts were crossed into the *Mhc* ^*10*^ background containing the *fln‐*Gal4 construct (http://flybase.org/reports/FBtp0097341) to drive *Mhc* expression.

### 4.4 Flight and jump assays

Transgenic lines were assayed for flight ability by determining upward (U), horizontal (H), downward (D), or no flight (N). Flight assays were performed at 22°C on ~100 flies for each transgenic line. Flight index was calculated as 6U/T + 4H/T + 2D/T + 0N/T, where T is the total number of flies tested. Flies were grouped into cohorts of 10–20, with each cohort average serving as a single data point.

Jump ability of 20 homozygous flies from each line was tested at 22°C. The greatest three jump distances out of 10 jumps per fly were averaged and used as a single data point.

### 4.5 Electron and light microscopy

Transmission electron microscopy was performed as previously described (O'Donnell & Bernstein, 1988). Cross‐ and longitudinal sections were obtained from females, with at least three different organisms examined for each transgenic line. For light microcopy, 1.0‐μm thick sections were taken from blocks prepared for electron microscopy.

### 4.6 Myosin extraction and purification

Dorsolongitudinal IFM were scraped from dissected female virgin flies (1‐3 days old). Myosin was extracted following methods previously described (Kronert et al., 2008; Swank et al., 2001) and detailed in Supplementary Materials.

### 4.7 Myosin ATPase assay

ATPase activity was measured by the malachite green colorimetric assay, as detailed in Supplementary Materials. Values for *V*_*max*_ and *K*_*m*_ were determined (after subtracting basal ATPase activity) by plotting actin‐activated myosin ATPase activity vs. actin concentration according to Michaelis–Menten kinetics.

### 4.8 In vitro motility assay

Actin sliding velocity arising from myosin interaction was determined by computational analysis of in vitro fluorescent optics videos. Nitrocellulose‐coated coverslips were used to assemble flow cells that were loaded with IFM myosin and rinsed with sheared unlabeled phalloidin‐stabilized F‐actin filaments. Addition of TRIT‐C phalloidin‐labeled actin filaments and ATP permitted viewing of filament movement.

### 4.9 Myosin filament forming assay

Myosin aliquots were diluted with NaCl of varying concentrations. Each sample was incubated on ice for 30 min, with monomeric myosins remaining in the supernatant and filaments in the pellet after centrifugation (Viswanathan et al., 2017). Pellets and supernatants were electrophoresed, and images of protein bands were quantified with UN‐SCAN‐IT software (Silk Scientific, v. 6.1).

### 4.10 Western blotting

Whole thoraces were dissected, shredded, and centrifuged to prepare extracted supernatant lysate. Following protein concentration determination and electrophoresis, samples were transferred to nitrocellulose and Ponceau S staining was performed. Blocked blots were treated with K‐48‐specific polyubiquitin rabbit monoclonal antibody (Cell Signaling Technology #8081) or anti‐Forkhead box protein O/dFOXO rabbit polyclonal antibody (Abcam #ab195977). Blots were washed and probed with goat anti‐rabbit secondary antibody conjugated to horseradish peroxidase, and chemiluminescence signals were quantified with UN‐SCAN‐IT software.

### 4.11 FOXO transgene overexpression

Overexpression of the *FOXO* transgene (#9575) via the *flightin‐Gal4* driver in the *N1168D‐2*/+ genotype tested for improved muscle function. Flies were grown at 20°C or at 25°C. Tested individuals had the genotypes: *fln‐Gal4, Mhc* ^*10*^ */UAS‐FOXO; N1168D‐2*/*−* (experimental line), *yw/w; Mhc* ^*10*^ */UAS‐FOXO; N1168D‐2*/*−* (UAS‐FOXO only control) and *yw/w; fln‐Gal4, Mhc* ^*10*^ */+; N1168D‐2*/*−* (*fln* driver only control), where “−” indicates no transgene.

### 4.12 Statistical analyses

For flight testing, female flies (in experiments that included X‐linked transgenes) or balanced quantities of male and female flies were grouped into cohorts of 10–20. For homozygotes, the mean flight index of all cohorts for a particular line was compared to those of the control with Welch and Brown‐Forsythe one‐way ANOVA. This approach was also used for comparing flight abilities of FOXO effects on *N1168D*/+ flies at 20 or 25°C at each particular age. For heterozygotes and *UAS* line flight as well as homozygote jump ability studied over time, a two‐way ANOVA with Dunnett's multiple comparisons tests was utilized. Slopes of changes in flight ability during aging were compared by simple linear regression. For in vitro motility, n > 25 myosin filament velocities were averaged for each biological replicate. Mean velocities from multiple biological replicates were compared to control by unpaired *t‐*tests with Welch's correction for unequal variances. For ATPase, nonlinear regression with a Michaelis–Menten curve fit provided reaction rates at increasing concentrations of actin. Basal ATPase, actin‐stimulated *V*_*max*_ and *K*_*m*_ were compared using unpaired *t‐*tests with Welch's correction for unequal variances. For filament formation assays, one‐way ANOVA with Dunnett's multiple comparisons test compared myosin filament formation at the EC50 (Viswanathan et al., 2017). For western blots, *pwMhc2* or *yw/w; fln‐Gal4, Mhc* ^*10*^ */+; N1168D/−* total protein pixels were used as normalization references. One‐way ANOVA with, respectively, Dunnett's or Sidak's multiple comparisons tests between each experimental ratio and the control (set to 1) compared differences in K48‐linked polyubiquitination or FOXO at each time point. Statistical analyses of *p* values, which were considered significant at *p* < 0.05, were performed using GraphPad Prism (GraphPad Software Inc., La Jolla, CA).

## AUTHOR CONTRIBUTIONS

Conception and design: TH and SIB. Acquisition of data: CLN, WAK, JRTC, and JAS. Analysis and interpretation of data: CLN, WAK, JRTC, TH, and SIB. Manuscript drafting: CLN, WAK, and SIB. Final manuscript approval: CLN, WAK, JRTC, JAS, TH, and SIB. Accountable for accuracy and integrity: CLN, WAK, JRTC, JAS, TH, and SIB.

## FUNDING INFORMATION

National Institutes of Health grant R37GM032443 and diversity supplement R37GM032443‐37S1; Rees‐Stealy Research Foundation‐SDSU Heart Institute Fellowship.

## CONFLICT OF INTEREST STATEMENT

The authors have no conflicts of interest to disclose.

## Supporting information


Data S1.

Appendix S1.

## Data Availability

All data are included within the manuscript, Supplementary Materials and Data Supplement.
