# Supplementary material for: Aging‐affiliated post‐translational modifications of skeletal muscle myosin affect biochemical properties, myofibril structure, muscle function, and proteostasis
Source: Aging Cell. 2024 Mar 20;23(6):e14134. doi: 10.1111/acel.14134 (PMC11296117; doi:10.1111/acel.14134)
Supplement: Supplementary file 2 — Appendix S1. [file ACEL-23-e14134-s001.pdf]

SUPPLEMENTARY MATERIALS

Aging-affiliated post-translational modifications of skeletal muscle myosin affect biochemical properties, myofibril structure, muscle function and proteostasis

Clara L. Neal, William A. Kronert, Jared Rafael T. Camillo, Jennifer A. Suggs, Tom Huxford and Sanford I. Bernstein

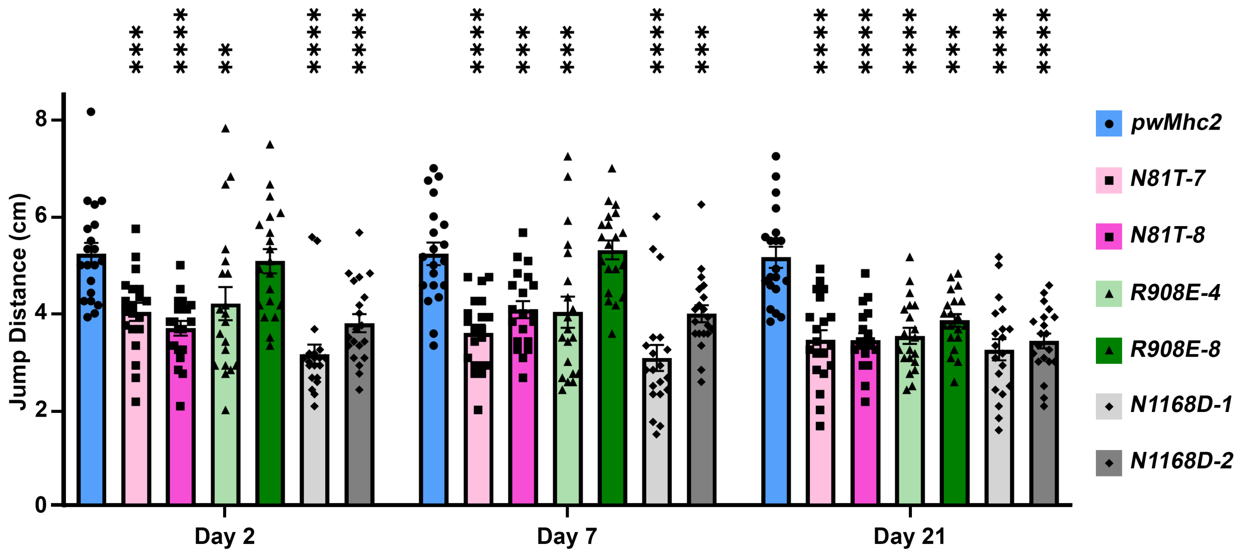

SUPPLEMENTARY FIGURE 1: Myosin PTM mimics that severely affect flight ability show negative effects upon jump muscle function in homozygotes during aging relative to control. All values are mean ± SEM (*=p<0.05, **= p<0.01, ***= p<0.001, ****= p<0.0001).

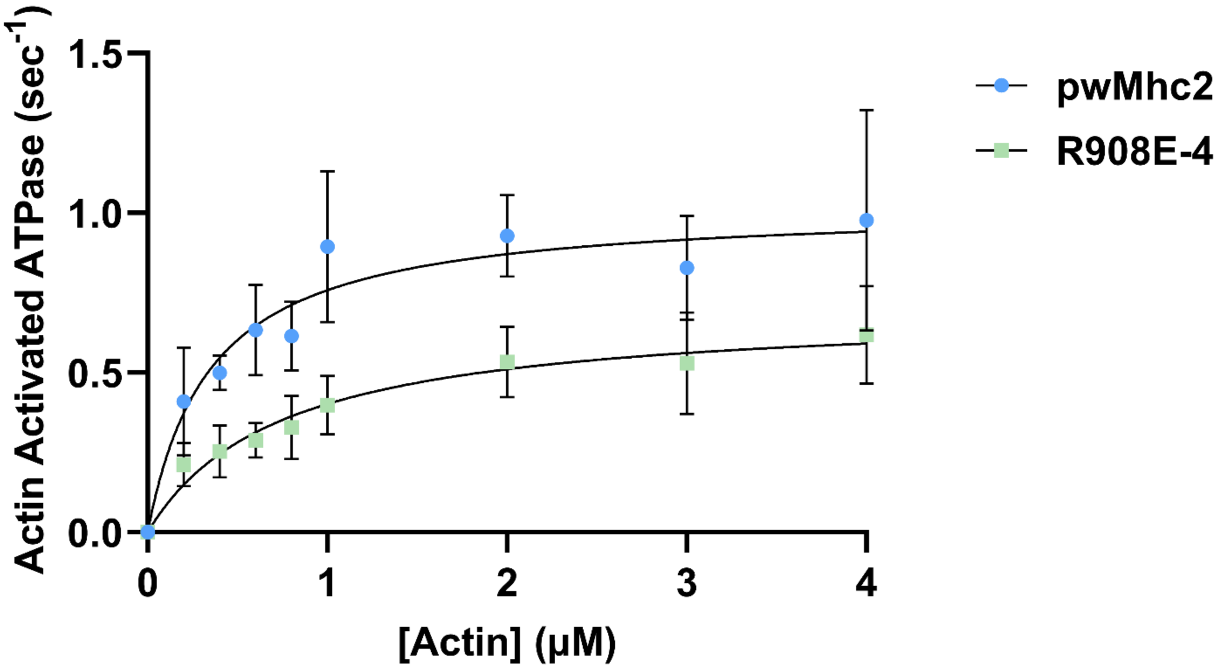

SUPPLEMENTARY FIGURE 2: Actin-activated Mg^{2+}-ATPase activity of pwMhc2 (wild-type) and R908E PTM-mimic myosin fit to the Michaelis-Menten equation following subtraction of basal activity. The V_{max} value for R908E is significantly reduced compared to wild-type (Table 2).

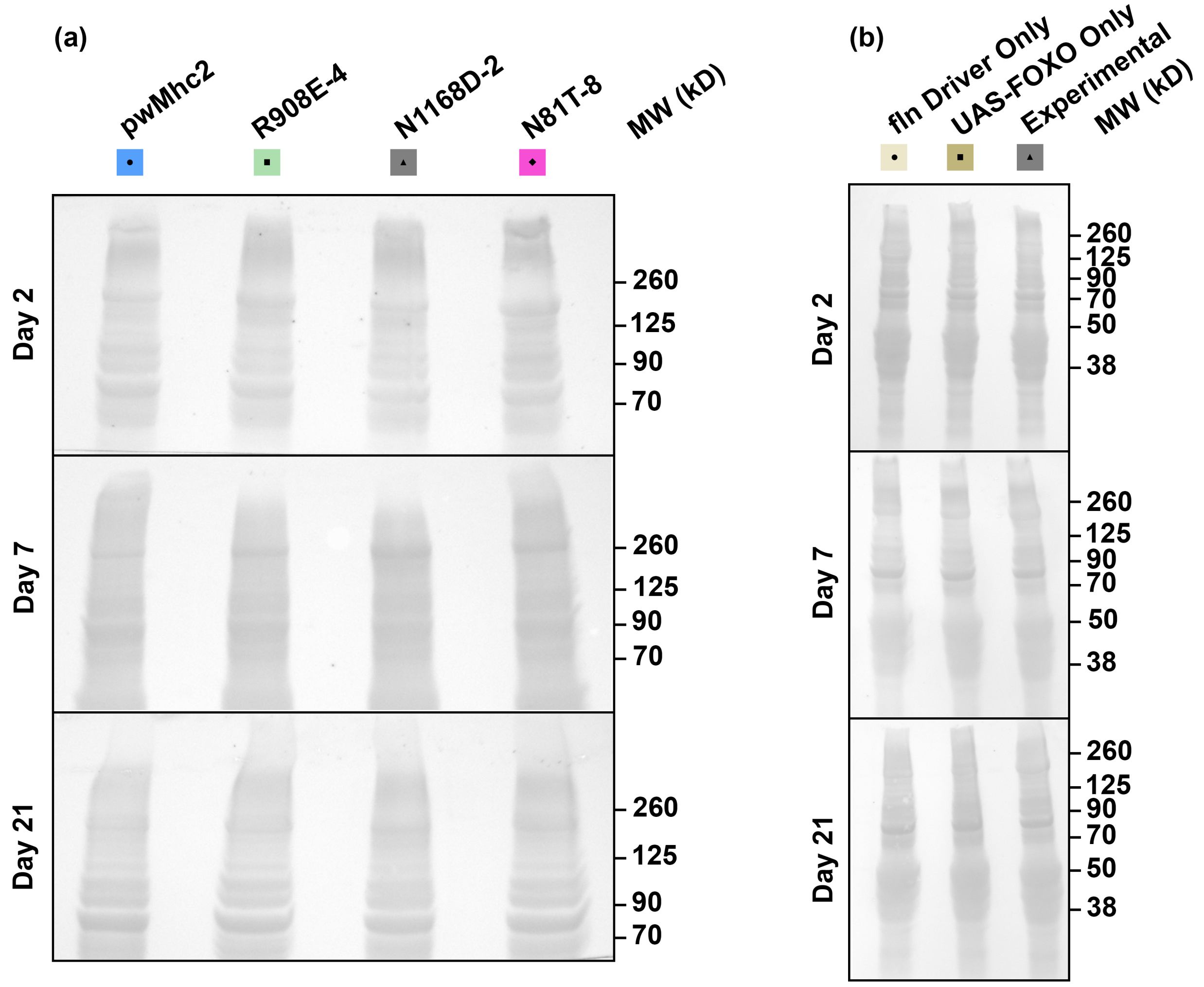

SUPPLEMENTARY FIGURE 3: Images of Ponceau stained western blots used for (a) K48-linked polyubiquitin detection or (b) FOXO detection as shown in Figures 7a-b and 7c-d, respectively. Pixel counts and calculation of normalized antibody levels are given in the Data Supplement for these samples and for biological replicates.

DETAILED EXPERIMENTAL PROCEDURES

DNA constructs

The *Drosophila P*-element-containing *Mhc* genomic construct *pwMhc2* (Swank et al., 2000) was digested with Eag I to produce two subclones. The *pwMhc-5'* subclone contains an 11.3 kb fragment in *pCasper*. The *pMhc-3'* subclone contains a 12.5 kb fragment in *pBluescriptKS* (Stratagene, La Jolla, CA). These served as substrates for site-directed mutagenesis.

For production of *N81T* and *N81A* mutant transgenes, *pwMhc-5'* was digested with Xho I and Avr II. A 6.8 kb fragment was gel isolated and ligated into *pLitmus 28I* (New England Biolabs). This subclone was digested with Pst I and Avr II to yield a 4.3 kb fragment that was ligated into *pLitmus 28I*. The resulting subclone was digested with Pst I and Age I, yielding a 1.7 kb fragment that was ligated into *pLitmus 28I*. The subsequent subclone was subjected to site-directed mutagenesis using the QuickChange II kit (Agilent) and primer 5'-TGCTCCAGCAAGTGACCCCCCCGAA -3' containing the N81T nucleotide coding change (underlined). For N81A, 5'- TGCTCCAGCAAGTGGCCCCCCCGAA -3' containing the N81A mutation (underlined) was used. The mutated subclone fragments were sequentially cloned back into the intermediate subclones from which they originated. The resulting plasmids were digested with Eag I. The 12.5-kb Eag I fragment of pMhc-3' was ligated into the mutated subclones, to yield *pwMhcN81T* and *pwMhcN81A*.

For R908A, R908E, R908M, N1168A and N1168D, the *pMhc-3'* subclone was digested with Sbf I and Stu I. A 2.7 kb Sbf I-Stu I fragment was ligated into *pLitmus 28I* and subjected to site-directed mutagenesis using the following primers:

5'- GGACTACCAGGAGGCTAACGCCAAGTTGAC -3' (R908A),

5'- GGACTACCAGGAGGAGAACGCCAAGTTGAC -3' (R908E),

5'- GGACTACCAGGAGATGAACGCCAAGTTGAC -3' (R908M),

5'- CCAGATTGAGCTCGCTAAGAAGCGTGAGGC -3' (N1168A), or

5'- CCAGATTGAGCTCGATAAGAAGCGTGAGGC -3' (N1168D). Upon sequence confirmation, each subclone fragment was sequentially cloned back into its parent subclone. The 12.5-kb Eag I fragment from each was then ligated into the Eag I site of *pwMhc-5'* to yield *pwMhcR908A*, *pwMhcR908E*, *pwMhcR908M*, *pwMhcN1168A* and *pwMhcN1168D*.

Mimic mutations N81T, R908E and N1168D were also cloned into the *pUASattB* vector for transgenic insertion using the PhiC31 integrase system (Bischof et al., 2007). To produce the *pUASattBMhc* control plasmid, a 4.7 kb PCR fragment was generated from the start of exon 2 (5' non-coding transcribed region) through exon 7d. The positive primer introduced Eco RI and Bsi WI restriction sites (underlined) at the 5' end of exon 2 (bold)

5’- CCGGAATTCCGTACG**GAAGTTTTGGGCTCACGACGC -3’.** A negative PCR primer in exon 7d (5’- GCTGGAATTCCTCACCGTCATCCATGTTGGGTAC -3’) contains a natural Eco RI site (underlined). Following PCR, the 4.7 kb Eco RI fragment was ligated into Eco RI-digested *pUASattB* and *pLitmus 28i* vectors to yield *pUASattB4.7RI* and *pLitMhc4.7RI*. *pUASattB4.7RI* was digested with Avr II and Eag I and a 4.4 kb fragment from *pwMhc5’* was ligated into it to yield *pUASattBMhc5’*. The 12.5-kb Eag I fragment from pMhc-3’ was ligated into this clone to yield *pUASattBMhc*. For introduction of N81T, a 1.7 kb Mfe I-Sgr AI fragment from *pwMhcN81T-5’* was ligated into *pLitMhc4.7RI*. A 4.4 kb Bsi WI-Avr II fragment from this subclone was ligated into the Bsi WI-Sgr AI site of *pUASattBMhc5’* to yield *pUASattBN81T-5’*. Ligation of the 12.5-kb Eag I fragment from *pMhc-3’* into *pUASattBN81T-5’* yielded *pUASattB-N81T*. For R908E and N1168D, the 12.5-kb Eag I fragment from *pMhcR908E-3’* or *pMhcN1168D-3’* was ligated into *pUASattBMhc5’* to yield *pUASattB-R908E* and *pUASattB-N1168D*. For all full-length clones, the entire coding region and all ligation sites were confirmed by DNA sequencing (Eton Bioscience, San Diego, CA).

Production of transgenic lines

One thousand embryos were injected for each *P* element-mediated transformation (Rubin & Spradling, 1982). Chromosome locations were mapped using balancer chromosomes and standard genetic crosses. Outcomes were as follows: *pwMhcN81A*: 2 lines on X chromosome, 9 on 2nd, 9 on 3rd; *pwMhcN81T*: 2 on 2nd, 10 on 3rd; *pwMhcR908A*:1 on 2nd, 2 on 3rd; *pwMhcR908E*: 1 on X, 3 on 2nd, 8 on 3rd; *pwMhcR908M*: 2 on X, 6 on 2nd, 11 on 3rd; *pwMhcN1168A*: 7 on 2nd, 6 on 3rd; *pwMhcN1168D*: 2 on 2nd, 2 on 3rd. Further analyses were performed on 2-3 independent lines for each transgene (Table 1). For *pUASattB* mimic lines, Bloomington Drosophila Stock Center #8622 was injected to direct the insertions to the third chromosome, with analysis performed on the resulting stocks: *pUASattBN81T* (2 lines): *pUASattBR908E* (1 line); *pUASattBN1168D* (2 lines) (Table 1). All analyzed transgenic lines were crossed into the Mhc^{10} background, which is null for myosin heavy chain in IFM and TDT muscle (Collier et al., 1990). Transgenic lines were generated by BestGene, Inc. (Chino Hills, CA).

Transgenic line validation

RT-PCR (New England Biolabs Protoscript cDNA synthesis kit) was used to confirm that the *Mhc* transcripts from each homozygous transgenic line were spliced correctly and contained the appropriate site-directed nucleotide changes. $LiCl_2$ extraction was employed to prepare RNA

from upper thoraces of two-day-old adult female transgenic flies (Becker et al., 1992). For cDNA synthesis, 3 μmol of reverse primer was mixed with 0.5 μg of RNA from each transgenic line. To assess splicing of alternative exons 3, 7 and 9, a reverse primer for exon 10 (5'-TCGAACGCAGAGTGGTCAT -3') and a forward primer for exon 2 (5'-TGGATCCCCGACGAGAAGGA-3') were used. For alternative exons 11 and 15, a reverse primer for exon 16 (5'- GGGTGACAGACGCTGCTTGGT -3') and a forward primer for exon 10 (5'- GTTCCCCAAGGCCTCCGATCA -3') were used. PCR was performed using 1 μl of cDNA and 3 μmol of each primer pair using the following conditions: 60 s at 94 °C, 30 cycles of: 30 s at 94 °C, 30 s at 55 °C and 2 min at 68 °C. RT-PCR products were sequenced by Eton Bioscience.

We determined myosin expression levels relative to actin accumulation for each homozygous transgenic line in an Mhc^{10} background by SDS polyacrylamide gel electrophoresis and densitometry (O'Donnell et al., 1989). For transgenic *pUASattB* lines, inserts crossed into the Mhc^{10} background were crossed with flies containing a *fln*-Gal4 construct (http://flybase.org/reports/FBtp0097341) that had been recombined onto the Mhc^{10} second chromosome, in order to drive *Mhc* expression. Upper thoraces from five two-day-old female flies were homogenized in 60 μl SDS gel buffer. Five μl of sample were loaded on a 9% polyacrylamide gel; this was repeated five different times, each time using a freshly prepared sample. Protein accumulation was determined using Coomassie blue stained gels that were digitally scanned and analyzed on NIH Image J software (https://imagej.nih.gov/ij/).

Flight and jump assays

Transgenic lines were assayed for flight ability by determining upward (U), horizontal (H), downward (D) or no flight (N) from a release point 20 cm high inside a Plexiglas box with a light source at the top (Drummond et al., 1991). Flight assays were performed at 22°C on ~100 flies for each transgenic line. Flight index was calculated as 6U/T + 4H/T +2D/T + 0N/T, where T is the total number of flies tested (Tohtong et al., 1995). Flies were grouped into cohorts of 10-20; each cohort average value served as a single data point.

The jump ability of 20 homozygous flies from each line was tested at 22°C after surgically removing wings from newly eclosed female flies and allowing a 48-hour recovery time. Flies were encouraged to jump from a pedestal 9.5 cm high by tapping a paint brush on its edge to cause vibration. The horizontal jump distance was measured and noted on a paper marked with concentric rings surrounding the pedestal (Eldred et al., 2010). The greatest three jump distances out of ten jumps per fly were averaged and used as a single data point.

Electron and light microscopy

Transmission electron microscopy was performed as previously described (O'Donnell & Bernstein, 1988). Cross- and longitudinal-sections were obtained from females, with at least three different organisms examined for each transgenic line. Myofibrils shown in each panel are representative of the population at that given developmental stage. For light microcopy, 1.0-µm thick sections were taken from blocks prepared for electron microscopy. Slides containing the thick sections were placed on an 80°C heating block to remove moisture. Sections were then stained with 1% of toluidine blue on the heating block for 20 s. Sections were then rinsed with water and allowed to air dry.

Myosin extraction and purification

Half thoraces were separated at 4°C from 100-200 virgin female flies with micro-scissors and dorsolongitudinal IFM were scraped from the cuticle using a curved wire hand tool. Following methods previously described (Kronert et al., 2008; Swank et al., 2001), fibers were transferred to York Modified Glycerol (YMG: 20 mM KPi, pH 7.0, 2 mM $MgCl_2$, 1 mM EGTA, 20 mM DTT, 50% v/v glycerol) containing a cOmplete™ Mini Protease Inhibitor Cocktail tablet (Roche). Following centrifugation (5 min, 15,000 x g, ~13000 rpm in a Beckman FA241.5P fixed angle rotor), the pellet was resuspended in YMG with 2% Triton-X detergent. The supernatant was discarded after another centrifugation (as above) and permeabilized fibers were washed free of detergent in YMG without glycerol so that myosin could be extracted for 15 min in 82.5 µl of myosin extraction buffer (1 M KCl, 50 mM KPi, pH 6.8, 5 mM $MgCl_{2,}$ 0.5 mM EGTA, 16.4 mM Na-pyrophosphate, 20 mM DTT, protease inhibitor tablet). The high salt extract was centrifuged (as previously) to remove insoluble material and myosin was precipitated on ice overnight after dilution to 40 mM KCl and 10 mM DTT. After centrifugation at 100,000 x g (43,000 rpm) for 20 min (Beckman TLA 100.3 fixed angle rotor), the pellet was dissolved in 13.5 µl Wash B (2.4 M KCl, 0.5 mM EGTA, 0.14 M histidine, 20 mM DTT, 90 mM KOH, protease inhibitor tablet) on ice for 30 min. Myosin remaining complexed with actin was precipitated by slowly adding 94.5 µL of 10 mM DTT to decrease the KCl concentration to 0.3 M. The sample was then centrifuged at 60,000 x g (33,300 rpm) for 25 min. The supernatant was removed, diluted 10-fold with 10 mM DTT, incubated on ice for 1 h and centrifuged at 100,000 x g (43,000 rpm) for 25 min. Following centrifugation, pelleted myosin was dissolved in myosin storage buffer (20 mM MOPS, 0.5 M KCl, 20 mM DTT, 2 mM $MgCl_2$) on ice for 30 min. The concentration of myosin was determined by spectrophotometry using the equation: [(A280-A310)/0.53] * dilution factor, where 0.53 is the

extinction coefficient for *Drosophila* myosin. Addition of myosin storage buffer allowed concentration adjustment as needed.

Myosin ATPase assay

ATPase activity was measured by monitoring moles of inorganic phosphate released per mole of myosin per s in the absence or presence of F-actin. F-actin was polymerized by combining 506 µL ACEX (2 mM Tris-HCl [pH 8.0], 0.2 mM $CaCl_2$, 0.2 mM ATP [pH 7.0], 1 mM dithiothreitol), 124 µL (169.6 µM) G-actin, and 70 µL 10X polymerization buffer (50 mM Tris-Cl [pH 8.0], 500 mM KCl, 20 mM $MgCl_2$, 10 mM ATP [pH 7.0]) for 20 min on ice. The tube was inverted before each use. F-actin ranging between 0.2-4 µM was added to 37.5 µL 4 mM ATP (pH 7.0) and 1.5 µL myosin isolated from IFM diluted to 0.75 µg/µL with MSB, mixed with 9.1 µL Mg^{2+} ATPase buffer (80 mM imidazole pH 6.0, 80 mM KCl, 8 mM $MgCl_2$, 0.8 mM $CaCl_2$). Reactions were incubated for 10 min. Upon immediate transfer to 500 µL 0.039% malachite green (in 1.1% ammonium molybdate, 1N HCl, and 0.0005% NP-40 alternative), binding of inorganic phosphate yielded green complexes (Littlefield et al., 2003). Each colorimetric reaction was stopped after 2 min with 50 µL of 34% sodium citrate, pH 2.0 (we note that 34% anhydrous citric acid, pH 1, stops the reaction most effectively). Controls included phosphate standards of 0-8000 pmol, a blank (no actin or myosin), and determination of actin ATPase activity without myosin. All samples were run in duplicate.

Spectrophotometer readings on 500 µL of malachite green reactions taken at A650 were averaged between technical replicates and interpolated within the standard curve using GraphPad Prism (GraphPad Software Inc., La Jolla, CA). Picomoles of inorganic phosphate generated by samples without myosin were subtracted from paired samples with myosin. Nanomoles of phosphate generated per min per µg of myosin were converted to the reaction rate in s^{-1}. Values for V_{max}, and K_m were determined (after subtracting basal ATPase activity) by plotting actin-activated myosin ATPase activity vs. actin concentration according to Michaelis-Menten kinetics.

***In vitro* motility assay**

Actin sliding velocity arising from myosin interaction was determined by computational analysis of *in vitro* fluorescent optics videos (Kron & Spudich, 1986). Nitrocellulose-coated coverslips for flow cells were prepared by putting ten drops of 1% nitrocellulose in amyl acetate (Ladd Research) on the surface of deionized water in a petri dish. After 10 s, coverslips were immersed on one side to bind nitrocellulose through water surface tension. They were dried

nitrocellulose-side up. Two 0.005 in thick, 1 mm wide Artus Motor Mount Shims were affixed to a clean microscope slide using Loctite 532 adhesive (Ellsworth Adhesives). When the adhesive is still malleable, the coverslip was pressed nitrocellulose-side down onto the shims. The resulting flow cell was placed under UV light for 20 min to cure the adhesive.

Flow cells were filled at a 45° angle with 50 µL of AB/BSA (870 µL H_2O, 20 µL 1 M DTT, 100 µL 10X AB [17.2 mg/ml Imidazole, 833 µL 3 M KCl, 400 µL 1 M $MgCl_2$, 1 mL 0.1 M EGTA, ~130 µL 6 M HCl to pH 7.4], 10 µL 50 mg/ml BSA). After flowthrough of a second 50 µL, the cell was incubated for 8 min. Subsequent additions to the flow cell were to maximum holding volume, unless otherwise noted. IFM myosin in MSB at a concentration 0.5 mg/ml was added and incubated for 2 min. Additional AB/BSA washed out unbound myosin. Flow cells were incubated for 1 min with 2 µM sheared unlabeled phalloidin-stabilized F-actin filaments (355 µL H_2O, 10 µL 1 M DTT, 45 µL 10X AB, 30 µL 30 µM F-actin, 0.5 µL 0.1 mg/ml phalloidin in MeOH) that had been stored on ice for 20 min and sheared by vortex immediately before use. A wash with AB/BSA removed unbound sheared F-actin. One volume of OB/MC/GOC (255 µL H_2O, 20 µL 1 M DTT, 25 µL SCAV mix [138 mg glucose, 1.1 mg catalase {ca 10750 U}, 5.5 mg glucose oxidase {ca 1550 U}, 600 µL H_2O added to dry mix before use], 700 µL of 20-30 min degassed (OB/MC [3 ml H_2O, 5 mL 0.8% methylcellulose, 900 µL 10X OB {same components as 10X AB, but no KCl, adjusted to pH 7.4}]) was added and solution was wicked off the end of the flow cell prior to addition of one volume of OB/MC/GOC/ATP (255 µL H_2O, 20 µL 1 M DTT, 25 µL SCAV mix, 700 µL of degassed OB/MC/ATP [3 mL H_2O, 5 mL 0.8% methylcellulose, 900 µL 10X OB + ATP {10X OB, 20 mM ATP, pH 7.4} degassed 20-30 min]) for a 10 min incubation. One volume of OB/MC/GOC, then three volumes of AB/BSA were added to wash out remaining ATP. Two volumes of 4 nM labeled F-actin filaments (876 µL H_2O, 20 µL 1 M DTT, 100 µL 10X AB, 5.5 µL labeled 1 µM F-actin filaments [376 µL H_2O, 10 µL 1 M DTT, 45 µL 10X AB, 15 µL 30 µM F-actin, 4.5 µL 100 µM TRIT-C phalloidin in MeOH {100 µM TRIT-C phalloidin dissolved in 1 mL MeOH; 13.5 aliquot is dried overnight and resuspended in 10 µL MeOH prior to use}]) were added and incubated for 3 min. One volume of AB/BSA washed out unbound, labeled F-actin. One volume of OB/MC/GOC was added and wicked from the flow cell. Labeled filaments were located in the field of view and recording at 10 frames per second captured motion initiated by addition of one volume of OB/MC/GOC/ATP. Images were captured on Piper software (Stanford Photonics) and the Fiji processing package of Image J (https://imagej.net/software/fiji/) was used to track smooth movements of the front end of filaments.

Myosin filament forming assay

Myosin was isolated from approximately 100 newly-eclosed female flies and 10 μg aliquots were diluted to 0.1 μg/μL with 100 μL NaCl of varying concentrations, yielding samples ranging from 500 mM to 50 mM. Each sample was incubated on ice for 30 min, with monomeric myosins remaining in the supernatant and filaments in the pellet after centrifugation at 100,000 x g (Beckman TLA 100.3 fixed angle rotor) for 30 min (Viswanathan et al., 2017). Each 100 μL supernatant fraction was added to 50 μL of 3X Laemmli sample buffer containing 75 mM DTT. Pellets were resuspended in 150 μL of 1X Laemmli sample buffer with 25 mM DTT. Samples were boiled at 95°C for 4-5 min. Ten μL of each sample were electrophoresed in polyacrylamide Tris-glycine 4-20% gradient gels (Bio-Rad Mini-PROTEAN TGX Gels) and stained overnight with Gel Code Blue (Thermo Scientific). The BioRad ChemiDoc XRS imager was used to capture images of the gels and protein band pixels were quantified with UN-SCAN-IT software (Silk Scientific, v. 6.1). Filaments formed in each fraction were taken as a ratio out of total myosin (pixels in pellet / [pixels in supernatant + pixels in pellet]) to extrapolate the percentage of myosin filaments formed at each NaCl concentration.

Western blotting

Whole thoraces were dissected, with wings and legs removed, from ≥ 24 female flies in 1X phosphate-buffered saline containing a cOmplete™ Mini Protease Inhibitor Cocktail tablet (Roche). Following centrifugation at 10,000 x g (Beckman FA241.5P fixed angle rotor) for 5 min and supernatant removal, 150 μL of RIPA buffer (Thermo Scientific™) with a protease inhibitor cocktail (5 mL 1X RIPA, 50 μL 200X PMSF [#8553 Cell Signaling Technology], 50 μL 100X Halt [Thermo Scientific™ Halt™ Protease and Phosphatase Inhibitor Cocktail]) was added to the pellet. Thoraces were shredded with a sonicator and centrifuged at 14,000 x g for 10 min to prepare the extracted supernatant lysate. Protein samples and standards were assessed as to concentration using the Pierce™ BCA Protein Assay Kit (Thermo Fisher). Concentrations of lysates in RIPA PIC and 4X Laemmli sample buffer were equalized to load 75 μg of each sample in a 15 μL polyacrylamide Tris-glycine 4-20% gradient gel (Bio-Rad Mini-PROTEAN TGX Gel). Protein was transferred to nitrocellulose in transfer buffer (795 mL H_2O, 100 mL 10X Tris-glycine Buffer no SDS, 5 mL 10X Tris-glycine Buffer with SDS, 100 mL MeOH) at 200 mA for 1 h. Ten min shaking in 0.1% Ponceau S (Sigma) [w/v] in 5% acetic acid [v/v] verified sufficient protein transfer and the blot was then rinsed with H_2O for 15 min. It was then placed in Antigen Pre-Treatment Solution (Thermo Super Signal Western Blot Enhancer) for 10 min. Blocking buffer (10 mL 5X PBST, 2.5 g milk powder, 40 mL H_2O, dissolved by vortex and strained through a coffee filter) was shaken over the blot for 1 h at room temperature. K-48-

specific polyubiquitin rabbit monoclonal antibody (Cell Signaling Technology #8081) was diluted 1:250 in Primary Antibody Diluent (Thermo Super Signal Western Blot Enhancer) and added to the blot at room temperature for 1 h. For the FOXO western blots, the same procedure was followed, except that the transfer buffer contained 799 mL H_2O, 100 mL 10X Tris-glycine Buffer no SDS, 1 mL 10X Tris-glycine Buffer with SDS and 100 mL MeOH. The anti-Forkhead box protein O/dFOXO rabbit polyclonal antibody (Abcam #ab195977) was diluted 3:500 in Primary Antibody Diluent and added to the blot at 4°C overnight. Blots were washed in 1X TBST (20 mM Tris, 100 mM NaCl, 0.2% Tween) for 30 min and probed with a 1:5,000 goat anti-rabbit secondary antibody conjugated to horse radish peroxidase (HRP) (Immun-Star Goat Anti-Rabbit (GAR)-HRP Conjugate #1705046) in blocking buffer for 1 h at room temperature. Following an additional TBST wash, HRP was activated with the Super Signal West Pico Plus Kit. Chemiluminescence was detected in a BioRad ChemiDoc XRS imager, with exposure times between 30-500 s. Pixels of K-48 linked ubiquitin signal, FOXO bands and of total protein (Ponceau S stain) were quantified with UN-SCAN-IT software (Silk Scientific, v. 6.1).

FOXO transgene overexpression

A *UAS-FOXO* fly line was obtained from the Bloomington Stock Center (#9575). Expression of *UAS*-linked transgenes occurs in a Gal4-dependent manner (Duffy, 2002), as Gal4 binds to the Upstream Activation Sequence (UAS) to activate expression of a target gene. Overexpression of the target transgene *FOXO* via the *flightin-Gal4* driver in the *N1168D-2*/+ genotype tested whether FOXO improves muscle function. Flies from the PTM line were grown at 20°C or at 25°C prior to flight testing to observe whether one FOXO expression level is optimal for improving muscle function. Elevated temperature enhances the activity of the Gal4/UAS system, and thus induces more FOXO expression (Duffy, 2002). Tested individuals had the genotypes: *fln-Gal4, Mhc^{10} /UAS-FOXO; N1168D-2*/- (experimental line), *yw/w; Mhc^{10}/UAS-FOXO; N1168D-2*/- (UAS-FOXO only control) and *yw/w; fln-Gal4, Mhc^{10}/+; N1168D-2*/- (*fln* driver only control), where "-" indicates no transgene present.

Statistical analyses

For flight testing, female flies (in experiments that included X-linked transgenes) or balanced quantities of male and female flies were grouped into cohorts of 10-20. For homozygotes, the mean flight index of all cohorts for a particular line was compared to those of the control with Welch and Brown-Forsythe one-way ANOVA. This approach was also used for comparing flight abilities of FOXO effects on *N1168D*/+ flies at 20 or 25°C at each particular age. For

heterozygotes and UAS line flight as well as homozygote jump ability studied over time, a two-way ANOVA with Dunnett's multiple comparisons tests was utilized. Slopes of changes in flight ability during aging were compared by simple linear regression. Jump and flight-testing results are reported as mean ± SEM. For *in vitro* motility, the velocities calculated with ImageJ Fiji software of n > 25 myosin filaments were averaged for each biological replicate. Mean velocities from multiple biological replicates for PTM mimics were compared to the pwMhc2 control myosin using unpaired *t* tests with Welch's correction for unequal variances. For ATPase, nonlinear regression with a Michaelis-Menten curve fit provided reaction rates at increasing concentrations of actin. These allowed calculation of actin-stimulated V_{max} and K_m. These values as well as basal Mg-ATPase were compared using unpaired *t* tests with Welch's correction for unequal variances to determine statistical significance. For filament formation assays, nonlinear regression with EC50 shift, where X is the concentration of salt, evaluated the percentage of myosin that formed filaments. One-way ANOVA with Dunnett's multiple comparisons test compared myosin filament formation at the EC50 (Viswanathan et al., 2017). For western blots, the pwMhc2 lane was used as the reference for normalization. The normalization factor of every lane was calculated by dividing the pixel quantification of the total protein from the pwMhc2 sample by the total protein quantification for each PTM (Aldridge et al., 2008). The normalized pixel value for K48-linked polyubiquitin for each fly line was found by multiplying the normalization factor by its K48-linked polyubiquitin pixel value. One-way ANOVA with Dunnett's multiple comparisons tests between each PTM mimic ratio and the pwMhc2 control set to 1 compared differences in K48-linked polyubiquitination at each time point. A similar approach was used to assess FOXO expression, with the reference lane being of the genotype *yw/w; fln-Gal4, Mhc^{10}/+; N1168D/-* and analysis with one-way ANOVA with Sidak's multiple comparisons tests. Statistical analyses of *p* values, which were considered significant at $p < 0.05$, were performed using GraphPad Prism (GraphPad Software Inc., La Jolla, CA).

REFERENCES

Aldridge, G. M., Podrebarac, D. M., Greenough, W. T., & Weiler, I. J. (2008). The use of total protein stains as loading controls: an alternative to high-abundance single-protein controls in semi-quantitative immunoblotting. *J Neurosci Methods*, *172*(2), 250-254. https://doi.org/10.1016/j.jneumeth.2008.05.003

Becker, K. D., O'Donnell, P. T., Heitz, J. M., Vito, M., & Bernstein, S. I. (1992). Analysis of Drosophila paramyosin: identification of a novel isoform which is restricted to a subset of adult muscles. *J Cell Biol*, *116*(3), 669-681. https://doi.org/10.1083/jcb.116.3.669

Bischof, J., Maeda, R. K., Hediger, M., Karch, F., & Basler, K. (2007). An optimized transgenesis system for Drosophila using germ-line-specific phiC31 integrases. *Proc Natl Acad Sci U S A*, *104*(9), 3312-3317. https://doi.org/10.1073/pnas.0611511104

Collier, V. L., Kronert, W. A., O'Donnell, P. T., Edwards, K. A., & Bernstein, S. I. (1990). Alternative myosin hinge regions are utilized in a tissue-specific fashion that correlates with muscle contraction speed. *Genes Dev*, *4*(6), 885-895. https://doi.org/10.1101/gad.4.6.885

Drummond, D. R., Hennessey, E. S., & Sparrow, J. C. (1991). Characterisation of missense mutations in the Act88F gene of Drosophila melanogaster. *Mol Gen Genet*, *226*(1-2), 70-80. https://doi.org/10.1007/bf00273589

Duffy, J. B. (2002). GAL4 system in Drosophila: a fly geneticist's Swiss army knife. *Genesis*, *34*(1-2), 1-15. https://doi.org/10.1002/gene.10150

Eldred, C. C., Simeonov, D. R., Koppes, R. A., Yang, C., Corr, D. T., & Swank, D. M. (2010). The mechanical properties of Drosophila jump muscle expressing wild-type and embryonic Myosin isoforms. *Biophys J*, *98*(7), 1218-1226. https://doi.org/10.1016/j.bpj.2009.11.051

Kron, S. J., & Spudich, J. A. (1986). Fluorescent actin filaments move on myosin fixed to a glass surface. *Proc Natl Acad Sci U S A*, *83*(17), 6272-6276. https://doi.org/10.1073/pnas.83.17.6272

Kronert, W. A., Dambacher, C. M., Knowles, A. F., Swank, D. M., & Bernstein, S. I. (2008). Alternative relay domains of Drosophila melanogaster myosin differentially affect ATPase activity, in vitro motility, myofibril structure and muscle function. *J Mol Biol*, *379*(3), 443-456. https://doi.org/10.1016/j.jmb.2008.04.010

Littlefield, K. P., Swank, D. M., Sanchez, B. M., Knowles, A. F., Warshaw, D. M., & Bernstein, S. I. (2003). The converter domain modulates kinetic properties of Drosophila myosin. *Am J Physiol Cell Physiol*, *284*(4), C1031-1038. https://doi.org/10.1152/ajpcell.00474.2002

O'Donnell, P. T., & Bernstein, S. I. (1988). Molecular and ultrastructural defects in a Drosophila myosin heavy chain mutant: differential effects on muscle function produced by similar thick filament abnormalities. *J Cell Biol*, *107*(6 Pt 2), 2601-2612. https://doi.org/10.1083/jcb.107.6.2601

O'Donnell, P. T., Collier, V. L., Mogami, K., & Bernstein, S. I. (1989). Ultrastructural and molecular analyses of homozygous-viable Drosophila melanogaster muscle mutants indicate there is a complex pattern of myosin heavy-chain isoform distribution. *Genes Dev*, *3*(8), 1233-1246. https://doi.org/10.1101/gad.3.8.1233

Rubin, G. M., & Spradling, A. C. (1982). Genetic transformation of Drosophila with transposable element vectors. *Science*, *218*(4570), 348-353. https://doi.org/10.1126/science.6289436

Swank, D. M., Bartoo, M. L., Knowles, A. F., Iliffe, C., Bernstein, S. I., Molloy, J. E., & Sparrow, J. C. (2001). Alternative exon-encoded regions of Drosophila myosin heavy chain modulate ATPase rates and actin sliding velocity. *J Biol Chem*, *276*(18), 15117-15124. https://doi.org/10.1074/jbc.M008379200

Swank, D. M., Wells, L., Kronert, W. A., Morrill, G. E., & Bernstein, S. I. (2000). Determining structure/function relationships for sarcomeric myosin heavy chain by genetic and transgenic

manipulation of Drosophila. *Microsc Res Tech*, *50*(6), 430-442. https://doi.org/10.1002/1097-0029(20000915)50:6<430::AID-JEMT2>3.0.CO;2-E

Tohtong, R., Yamashita, H., Graham, M., Haeberle, J., Simcox, A., & Maughan, D. (1995). Impairment of muscle function caused by mutations of phosphorylation sites in myosin regulatory light chain. *Nature*, *374*(6523), 650-653. https://doi.org/10.1038/374650a0

Viswanathan, M. C., Tham, R. C., Kronert, W. A., Sarsoza, F., Trujillo, A. S., Cammarato, A., & Bernstein, S. I. (2017). Myosin storage myopathy mutations yield defective myosin filament assembly in vitro and disrupted myofibrillar structure and function in vivo. *Hum Mol Genet*, *26*(24), 4799-4813. https://doi.org/10.1093/hmg/ddx359
